# Seed germination thermal niche differs among nine populations of an annual plant: A modeling approach

**DOI:** 10.1002/ece3.9240

**Published:** 2022-08-26

**Authors:** Keyvan Maleki, Carol C. Baskin, Jerry M. Baskin, Mohadeseh Kiani, Iraj Alahdadi, Elias Soltani

**Affiliations:** ^1^ Department of Agronomy and Plant Breeding Sciences, College of Aburaihan University of Tehran Tehran Iran; ^2^ Department of Biology University of Kentucky Lexington Kentucky USA; ^3^ Department of Plant and Soil Sciences University of Kentucky Lexington Kentucky USA

**Keywords:** delayed germination, germination synchrony, germination timing, physiological dormancy

## Abstract

Germination timing is an important determinant of survival and niche breadth of plants. The annual plant *Nigella sativa* occurs in diverse environments along a steep temperature gradient and thus is a suitable model for the study of germination behavior in response to temperature. We used a modeling approach to compare the germination thermal niche of seeds of nine populations of *N. sativa* produced in a common garden. Germination time courses were obtained by a newly developed process‐based model, and thermal niche was visualized by plotting germination breadth as a function of after‐ripening time. Seeds were sampled five times: immature (2 weeks before maturity), mature, and afterripened for 1, 2, and 5 months. Immature and mature seeds had a greater depth of dormancy than afterripened seeds, as estimated by lower values of high‐limit temperatures (*T*_h_). Afterripening increased germination percentage, synchrony, and thermal niche breadth of all nine populations. The highest asynchrony was for immature and mature seeds, and afterripening enhanced synchrony. Based on the new graphical method, *N. sativa* has Type 1 nondeep physiological dormancy, and thus, the germination niche is narrow at seed maturity, leading to a delayed germination strategy that is highly dependent on thermal time accumulated during afterripening. Our findings show that there is considerable variation in the germination thermal niche among populations. Temperature regimes in the natural habitats of *N. sativa* have played a significant role in shaping variation in thermal niche breadth for seed germination of this annual species. The models used in our study precisely predict germination behavior and thermal niche under different environmental conditions. The germination synchrony model also can estimate germination pattern and degree of dormancy during the year, suggesting a useful method for quantification of germination strategies.

## Graphical abstract

We used a modeling approach to compare the germination thermal niche of seeds of nine populations of *Nigella sativa* produced in a common garden. Germination time courses were obtained by a newly developed process‐based model, and thermal niche was simulated via a new graphical method.

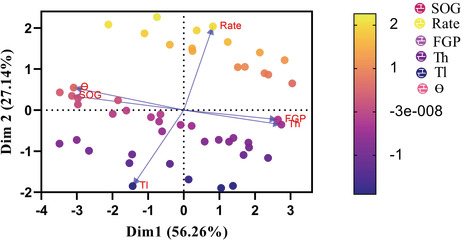

## 1 INTRODUCTION

Environmental variation is expected to become more unpredictable as a result of global warming. In response to climate change, understanding how organisms, particularly plants because of their sessile life style, deal with these stochastic environments is critical (Gremer et al., 2020). Ecological responses to both within‐ and among‐year fluctuations in environmental parameters, including temperature and precipitation, provide insight into how some organisms are adapted to unpredictable environments and how they take advantage of adaptative strategies (Bradshaw & Holzapfel, 2006; Diffenbaugh & Field, 2013; Norberg et al., 2012). At the plant level, changes in climatic patterns may have significant influences on ecological functions such as germination behavior (Gremer et al., 2020), growth, flowering pattern, and fruiting pattern. Some plants deal with unpredictability by hedging their bets, a strategy that reduces fitness variance at the expense of the arithmetic mean, (e.g., Gremer et al., 2016; Simons, 2011), whereas others use alternative strategies (Starrfelt & Kokko, 2012; ten Brink et al., 2020) such as a delayed‐germination risk‐avoidance strategy and a risk‐spreading strategy (Baskin & Baskin, 2014; Gremer et al., 2016, 2020). In a risk‐avoidance strategy, germination is delayed until environmental conditions are suitable for seedling establishment, while in a risk‐spreading strategy, that is, a bet‐hedging strategy, only a portion of the seed lot germinates, thereby spreading the risk of uncertainty of favorable conditions for seedling establishment across years (Gremer et al., 2016, 2020; ten Brink et al., 2020).

A key goal in ecology is to understand how a plant times its developmental events. In randomly changing environments, plants have various responses to seasonal environmental signals that coincide with germination, emergence, growth, and reproduction in suitable conditions (Andrés & Coupland, 2012; Blackman, 2017; Cohen, 1967; Gremer et al., 2016). Timing of developmental transitions may have effects on demographic, morphological, and physiological traits at each life history stage (Galloway & Burgess, 2009) and on the adaptive value of these traits (Donohue, 2002; Galloway & Burgess, 2009). For plants in a stochastic environment, the long‐standing question of when to germinate is a fundamental life history attribute with considerable impact on the life cycle (Burghardt et al., 2016; Galloway & Burgess, 2009). The conditions in which a newly emerged seedling survives and the likelihood of successful seedling establishment may be determined by germination timing (Postma & Agren, 2016). Depending on the time of germination, seedlings have different environmental niches, which are shaped by differences in length of growing season and resource availability and competition (Donohue et al., 2010).

Germination is a critical stage in plant life history that plays a vital role in plant establishment and in determining suitable conditions in which other life history events can take place (Baskin & Baskin, 2014; Gremer et al., 2020). Seed germination and seedling emergence, regarded as the most sensitive phases to environmental stochasticity, can influence individual fitness, population persistence, and the distribution of species (Baskin & Baskin, 2014). Among environmental drivers, temperature and water availability are of great importance since such environmental factors can act as seasonal cues sensed by plants (Huang et al., 2016). Many plants may have specific thermal and moisture requirements for germination, and that global climate change may pose the risk of nonfulfillment to these requirements (Baskin & Baskin, 2014).

Germination timing is an important determinant of population dynamics and species distribution (Clauss & Venable, 2000). In annual plant communities, adaptive predictive germination can be reflected by a difference in germination timing both within and among years, leading to germination in favorable conditions (Gremer et al., 2016; Simons, 2011). Plants have both spatial (dispersal syndromes) and temporal (seed dormancy) patterns that time germination and determine the environmental niche they experienced later in the life cycle, thus avoiding risky environments (Baskin & Baskin, 2014). Several studies have shown that phenology is highly dependent on germination strategy, seed dormancy, survival, and reproduction in response to weather conditions shifting across years (Donohue et al., 2015; Gremer et al., 2020). Of the phenology components correlated with survival, dormancy patterns and germination strategies (i.e., delayed germination) set the context for promoting survival after dispersal, and both are ecologically and evolutionary important (Baskin & Baskin, 2014).

There are five *classes* of dormancy: physiological (PD), morphological (MD), morphophysiological (MPD), physical and physical plus physiological (combinational) (Baskin & Baskin, 2004, 2014). Three levels of PD (nondeep, intermediate, and deep) have been identified, and nondeep PD is the most common kind of dormancy in seeds worldwide (Baskin & Baskin, 2014). Furthermore, there are six types of nondeep PD that fine‐tune the germination niche of species in response to temperature (Baskin & Baskin, 2014; Soltani, Baskin, et al., 2017). Of the six types of nondeep PD, three exhibit conditional dormancy during the dormancy‐breaking process (Soltani, Baskin, et al., 2017), in which the thermal range permissive for germination, and thus the thermal niche for germination, widens as seeds pass from dormancy to nondormancy (types 1, 2 and 3; Figure 2) (Baskin & Baskin, 2014; Batlla & Benech‐Arnold, 2015; Soltani, Baskin, et al., 2017). In species with conditional dormancy, the thermal niche breadth is limited and highly dependent on dormancy status, whereby a widening and narrowing of the thermal niche is likely to occur in response to dormancy release and dormancy induction, respectively (Batlla & Benech‐Arnold, 2015; Soltani, Baskin, et al., 2017; Soltani, Gruber, et al., 2017).

Afterripening, the release of PD with time during dry storage, may have a marked influence on variation in germination timing (Baskin & Baskin, 2020; Holdsworth et al., 2008). Dormancy loss due to afterripening is accompanied by a considerable number of changes in seed population sensitivity to environmental cues following imbibition (Alvarado & Bradford, 2005). The importance of afterripening in the adaptation of annual plants to periodic drought conditions is that it prevents premature germination during erratic timing of rainfall (Baskin & Baskin, 2014). Furthermore, afterripening may act as the functional link synchronizing germination strategies and plant life history in varying environments (Huang et al., 2016).

The concept of the thermal models widely used in plant development/phenology studies is that developmental units are accumulated over time in relation to the accumulated thermal units. Thus, population sensitivity to a given environmental condition can change, and seeds progressively gain the ability to germinate in response to seasonal environmental cues, thereby leading to evolution of the germination niche (Donohue et al., 2015; Maleki et al., 2021). The characteristics of the germination patterns under a wide range of environmental conditions can be mathematically described by population‐based models (PBT) using the normal distribution of parameters within a given population (Bello & Bradford, 2016; Bradford, 2018). Such models can define not only the threshold‐type response of a population (average behavior) but also the variability among individuals in dormancy status and germination timing within a population. These are important components of an opportunistic versus conservative strategy, and they set the context for subsequent developmental events (Batlla & Benech‐Arnold, 2015; Bello & Bradford, 2016; Bradford, 2018; Donohue et al., 2015).

At the population level, seed germination is a complex process due to the various dimensions involved in it, such as optimal conditions for germination, number of germinable seeds in a given population and synchrony and percentage of germination. Thus, characterization of germination is difficult, and several models have been developed to predict germination responses to factors regulating it, including temperature and moisture (Batlla & Benech‐Arnold, 2015; Donohue et al., 2015; Maleki et al., 2021; Soltani et al., 2022; Soltani et al., 2021).

This study compared breadth of the germination thermal niche among nine populations of *Nigella sativa* along a steep temperature gradient. We addressed the following questions about this annual species. (1) Are seeds conditionally dormant at maturity, and what type of dormancy do they have? (2) How does the germination thermal niche change with after‐ripening time? (3) Is germination of fresh seeds asynchronous within populations, and does synchrony increase with after‐ripening time? Our results illustrate not only the relationship between germination strategies and changes in temperature requirements for germination during afterripening but also provide a quantitative framework for predicting the germination niche in response to regional climate conditions. This study provides a novel perspective on correlation of germination, dormancy patterns, and thermal niche with long‐term mean temperatures for different populations of *N. sativa*.

## 2 MATERIALS AND METHODS

### 2.1 Study species

*Nigella sativa* (Ranunculaceae) is a winter annual distributed naturally in the Mediterranean region, middle Europe, and western Asia (Al‐Jassir, 1992). It has white to purple flowers and a capsule‐like fruit composed of up to seven follicles containing small black seeds (1–5 mg) that have an aromatic odor and taste (Al‐Jassir, 1992). *N. sativa* reaches maturity in late spring and early summer, depending on conditions. This species occurs in diverse environments along a steep temperature gradient (Table S1), providing a suitable model for studies on germination behavior in response to temperature. Variation in temperature is considerable during summer and may influence seasonal germination and breadth of the thermal niche. Seeds of *N. sativa* germinate during autumn to spring in the wild, depending on the climate conditions. Moreover, lack of uniform germination and seedling emergence among focal population of *N. sativa* (M. Kiani; Personal observation) means that additional research is required to advance our understanding of germination ecophysiology of this economically valuable species.

Unlike the mature seeds of many species of Ranunculaceae, which have an underdeveloped embryo and thus MD or MPD (Baskin & Baskin, 2014), those of *N. sativa* have a fully developed embryo (Butuzova et al., 1997) and nondeep PD (B_1_ sensu Nikolaeva et al., 1985). Thus, although the embryo is relatively small (embryo length: seed length ratio = 0.5) at seed maturity, it does not exhibit further growth inside the mature seed before the radicle emerges (Butuzova et al., 1997; K. Maleki, C. Baskin, J. Baskin, M. Kiani, I. Alahdadi, E. Soltani, unpublished data). Moreover, several authors have reported that *N. sativa* seeds are dark sensitive, that is, germinate better in dark than in light (Ahmadian et al., 2015; Alshammari, 2017; Nikolaeva et al., 1985). However, to mimic the natural environments in which *N. sativa* grows (Table S1), a daily 12‐h light/12‐h dark regime was used and in our study seeds germinated to very high percentages in a daily 12‐h light/12‐h dark regime (Figures S1–S5 see below).

### 2.2 Common garden

The starting populations of *N. sativa* included in this study were from seeds collected at nine locations that differ in geographic position, climate, and elevation (Table S1). These populations occur in fields and grassland throughout Iran. This species occurs across a wide elevational range (200–2000 m), and populations are discontinuously distributed. To eliminate maternal environmental effects on population differences in germination and thus to address variation in the seed germination thermal niche among nine populations of an annual plant, seeds for germination experiments were produced in a common garden. Seeds collected from the nine field populations were used to propagate plants in a common garden, and seeds from offspring of each population then were used to define the germination thermal niche. These seeds were produced in fairly well‐watered conditions, which can affect germination responses. For seeds used in germination experiments, we collected seeds produced in the common garden in late spring (2018) and allowed them to afterripen for up to 5 months. Germination tests were carried out immediately on immature and mature seeds after sampling, and the remaining seeds were stored dry in an incubator (20°C, darkness, ambient humidity, that is, 55%–60% relative humidity) prior to beginning the experiments.

**FIGURE 1:**
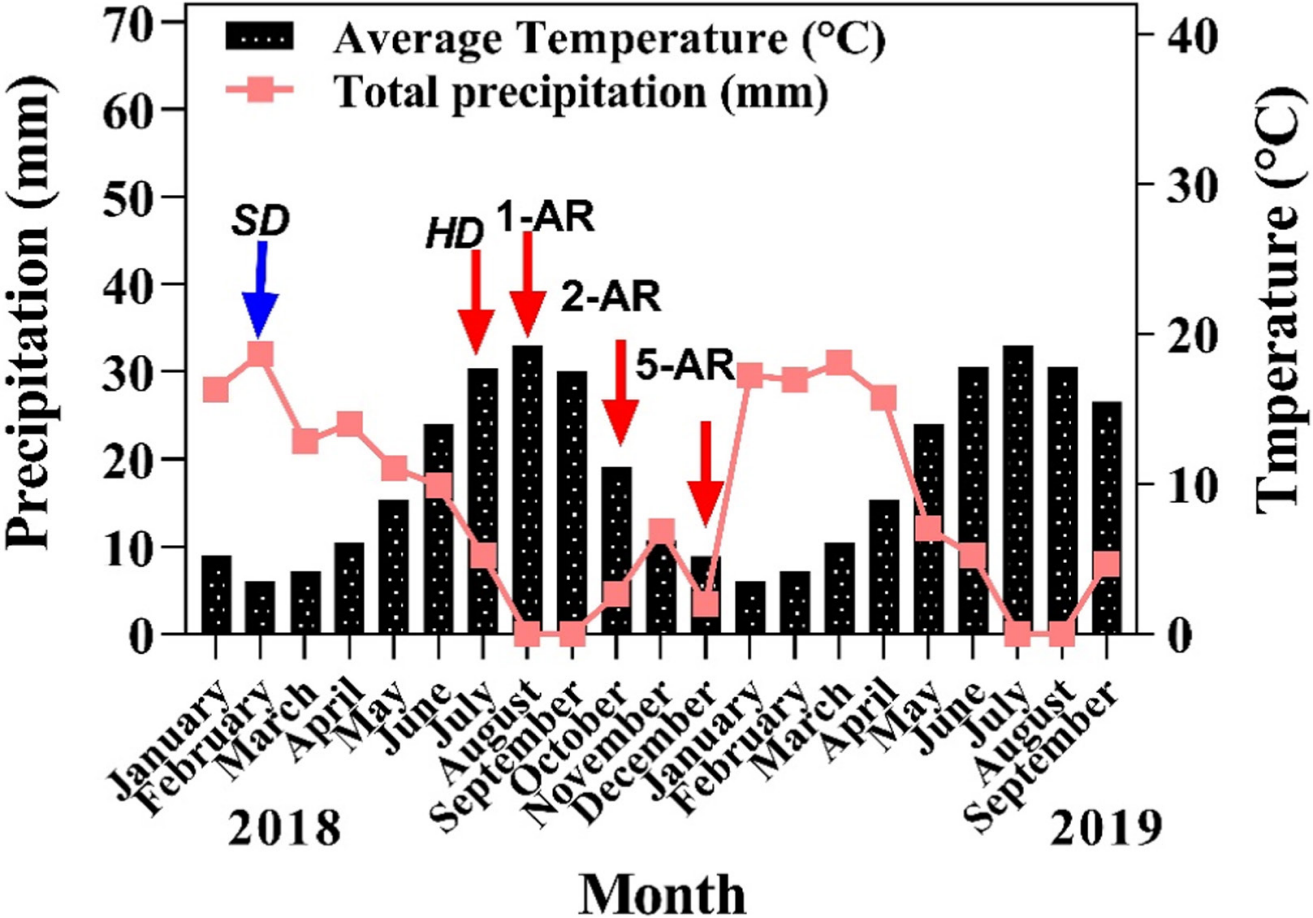
Average temperature and precipitation during experiments. The sowing date in common garden experiment and harvesting date were indicated in the panel. 1‐AR, one month of afterripening; 2‐AR, two months of afterripening; 5‐AR, five months of afterripening; HD, harvesting date; SD, sowing date.

The common garden is located on the Aburaihan Campus, University of Tehran (35°28′N, 51°36′E; 1020 m a.s.l.) in an area classified as an arid region according to the de Martonne climate classification (Raziei, 2017). The experiment was carried out in a randomized complete block design in 2018. Nine populations of *N. sativa* were cultivated in 27 transects in the common garden. Each transect was 1.5 m × 2 m, and the spacing between transects was 25 cm. We sowed seeds in the garden and then thinned the plants to a density of 120 m^−2^. Seeds were planted in late February, when both soil moisture and temperature are suitable for germination and growth (Kiani et al., 2020). Plants were watered at 10‐day intervals during winter and early spring and at 5‐day intervals in late spring and early summer to avoid any water stress. Populations reached maturity in late July. We harvested seeds when the capsules were brown, and all transects were harvested simultaneously to eliminate the differences in harvesting time and obtain seeds which are at the same developmental stage. Although maturity time was different among populations due to geographical origin and maternal environments, this difference was not considerable, so that we were able to harvest seeds simultaneously. Immature seeds (D) were harvested two weeks before maturity (M). Most of the annual precipitation occurs during winter and early spring. The lowest and highest temperatures occur in February and August, respectively (climatic data were extracted from NOAA's Climate Data Online Summaries Research Tool; https://www.ncdc.noaa.gov/climate‐information) (Figure 1).

### 2.3 Dormancy status of immature, mature, and after‐ripened seeds

To determine whether seed dormancy is expressed during different developmental stages, we intentionally harvested seeds at two intervals (1) two weeks prior to maturity (immature seeds, D) and (2) at maturity (mature seeds, M). After seed collection (at maturity), the seeds were air‐dried and winnowed to eliminate light‐weight seeds and empty fruits. Then, the seeds were stored in paper bags at constant 20°C in darkness for 1, 2, and 5 months for after‐ripening treatments.

### 2.4 Germination trials

Germination tests were carried out on immature (D), mature (fresh; M), and after‐ripened (1, 2 and 5 months) seeds with three replicates of 20 seeds for each treatment. The germination tests were grouped into five distinct experiments with a gradient of six temperatures from 5 to 30°C in 5°C intervals for all populations. The experiments were performed at five distinct intervals, including two weeks prior to maturity, at maturity and after 1, 2, and 5 months of afterripening. Seeds derived from all nine populations failed to germinate at 30°C; thus, this temperature was removed from both the model and the data analyses.

Germination was assessed in alternating light and darkness (12 h/12 h). The light source was cool white fluorescent tubes, and photon flux density at shelf level was about 23 μmol m^−2^ s^−1^, 400–700 nm. This light/dark interval was used as 12 h of light are within the range of photoperiods that seeds are exposed to in the field. Seeds were incubated on two filter papers moistened with 7 ml of distilled water in 9‐cm‐diameter Petri dishes. For each population and germination trial, 20 seeds per Petri dish and three replicate dishes were used. The water in Petri dishes was replenished as needed. After each counting, we changed the position of each Petri dish to minimize position effects. Germination was scored daily, and germinated seeds (radicle protrusions ≥2 mm) were removed from the dishes.

### 2.5 Quantification of thermal niche using population thermal parameters

We quantified thermal parameters describing degree of dormancy using the model proposed by Batlla and Benech‐Arnold (2015). In the model, the fraction of germinated seeds (GF(*T*)) for each population (immature (D), mature (M), and afterripened (AR1, AR2, and AR5)) at different incubation temperatures was estimated using the following model (see Supporting Information for additional information).

(1) $$\mathrm{GF}\left(T\right)=\left\{\Phi \left[\left(T-T_{l\left(50\right)}\right)/\sigma_{\mathrm{Tl}}\right]-\left\{1-\Phi \left[\left(T-T_{h50}\right)/\sigma_{\mathrm{Th}}\right]\right\}\right\}$$

where Φ is the normal probability integral l, and $T_{l\left(50\right)}$ and $T_{h\left(50\right)}$ are mean lower limit and higher limit temperatures, respectively, with standard deviations of $\sigma_{\mathrm{Tl}}$ and $\sigma_{\mathrm{Th}},$ respectively. The two descriptive parameters ($T_{l\left(50\right)}$, and $T_{h\left(50\right)}$ are mean lower limit and higher limit temperatures) explain two bounds showing thermal niche, which is contingent on depth of dormancy. Degree of dormancy is interpreted by changes in temperature range permissive for germination. Germination will occur when the prevailing temperatures overlap with (or exceed) the permissive temperature parameters explained by the two descriptive limits, and the permissive thermal range varies within a population, consistent with the concept that individual seeds have different degrees of dormancy. Based on the degree of nondeep PD, a wide range corresponds to a low degree of dormancy and a narrow range to a high degree of dormancy. A thermal niche for germination will be established between the low‐ and high‐limit temperature changes (Arana et al., 2016; Batlla & Benech‐Arnold, 2015; Maleki et al., 2021). The estimated population‐based parameters (lower and higher limit temperatures) and thermal time required for germination (accumulated developmental units, discussed in Section 1) are assumed to be normally distributed within the seed population with a mean (*θ*_(50)_) and a standard deviation (*σ*_*θ*_), while the cardinal temperatures for germination, base temperature (*T*_b_), optimum temperature (*T*_o_), and maximum temperature (*T*_m_), are considered to be constant for the whole seed population (Arana et al., 2016; Batlla & Benech‐Arnold, 2015; Maleki et al., 2021).

Differential thermal time requirement for germination can define differences in germination timing among populations. In the suboptimal thermal range, thermal time required for germination is accumulated above *T*_b_, and each population requires a different amount of thermal time for the completion of germination. Thermal time between optimum temperature (*T*_o_) and maximum temperature (*T*_m_) (in the supra‐optimal thermal range) is accrued below the maximum temperature (*T*_m_), and it is assumed that thermal time remains constant for each seed in the population (variations in *T*_m_ reflect germination timing). Thermal time in the suboptimal thermal range (*θ*_sub,_ between *T*_b_ and *T*_o_) and the supra‐optimal thermal range (*θ*_sup_, between *T*_o_ and *T*_m_) can be calculated for different fractions of the population (*g*) with Equations (2) and (3), respectively (Arana et al., 2016; Batlla & Benech‐Arnold, 2015; Maleki et al., 2021) (see Supporting Information for additional information):

(2) $$\theta_{\mathrm{sub}}\;\left(g\right)=\left(T-T_{b}\right)\;t_{g}$$

(3) $$\theta_{\sup}\;\left(g\right)=\left(T_{m}-T\right)\;t_{g}$$

where *t*_*g*_ is the time required for completing germination of fraction *g* of the population. The model assumes that the base (*T*_b_) and ceiling (*T*_max_) temperatures for germination do not change within a population, when seeds become nondormant. We first estimated cardinal temperatures for all populations and then used the estimated parameters (*T*_b_ and *T*_max_) for fitting the model to our data (see Table S2).

Based on the above concept that thermal niche can be quantified via population‐based models having ecologically meaningful parameters, the following issues were taken into account.

1. We estimated seed germination parameters (i.e., *T*_b_, *T*_o,_ and *T*_max_) for *N. sativa* via temperature functions. These parameters are for nondormant seeds, and they are estimated via a temperature function (Table S2, see Supporting Information for additional information).
2. Population‐based parameters describing the thermal range permissive for seed germination for different after‐ripening time intervals for nine populations. This was made through an optimization procedure using excel “solver” in which model equations were adjusted to germination time‐course at 5, 10, 15, 20, and 25°C. The thermal time required for seed germination also was derived by optimization.
3. We developed a thermal‐time model for dormancy release and a preliminary model for afterripening.

### 2.6 Parameter estimation

We used a nonlinear optimization procedure to estimate population thermal parameters to determine thermal niche and degree of dormancy. In this procedure, the process of optimizing the root‐mean‐square error (RMSE) between simulated and germination time‐courses was conducted by varying population thermal parameters with the “Solver” plug‐in (Microsoft Excel) based on a generalized reduced gradient (GRG) nonlinear optimization (Lasdon et al., 1978). Once we had made the best estimates of population thermal parameters, the best‐fitting lines between simulated and germination time‐courses were obtained.

### 2.7 Thermal after‐ripening time model (TAR)

To quantify dormancy loss during afterripening, we established a relationship between changes in higher temperature limit ($T_{h50}$, see Section 2.5) and thermal after‐ripening time. This concept assumes that the widely fluctuating high‐temperature limit attained during dry storage can be considered as a linear function of temperature above a base temperature. The thermal history that seeds perceive is calculated as the sum of hourly temperature values in the seed zone (in our case, storage at 20°C). We used the following equation to integerate thermal after‐ripening time (Allen et al., 2007):

(4) $$\theta_{\mathrm{AT}}=\left(T_{s}-T_{\mathrm{sl}}\right)t_{\mathrm{ar}}$$

where *θ*_AT_ denotes the thermal time required for afterripening, *T*_s_ the storage temperature, *T*_sl_ the lower limit storage temperature below which afterripening does notoccur (the value is 5°C for *N. sativa*, data not shown) and tar the actual time in storage required for completion of afterripening (time required for $T_{h50}$ to change from its initial [D and M] to its final value [after‐ripening intervals]).

### 2.8 Synchrony of germination

**FIGURE 2:**
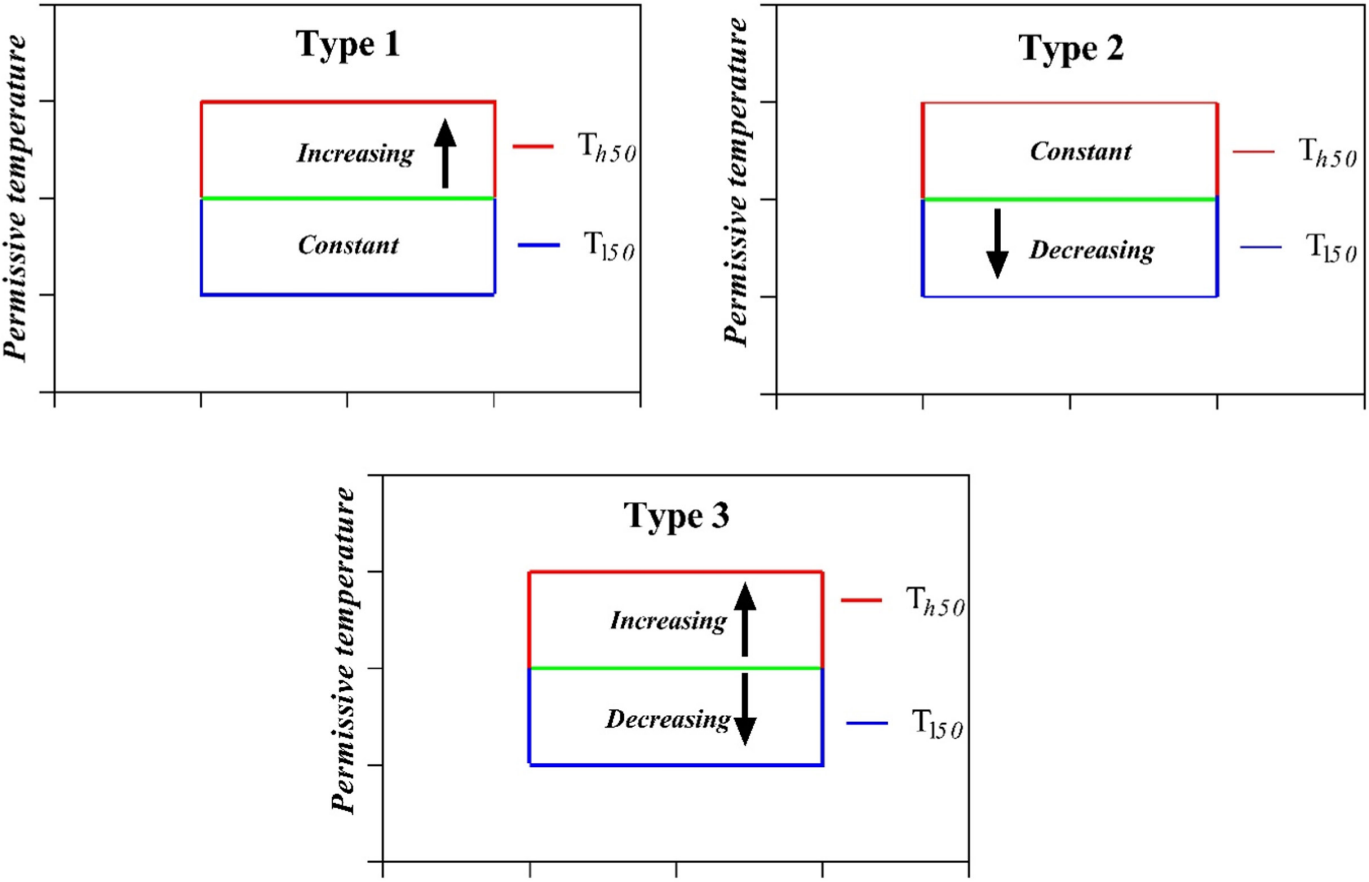
Three patterns (types 1, 2, and 3) of temperature requirements for germination of species with nondeep physiological dormancy as thermal niche changes. In types 1 and 2, dormant seeds germinate only at low ($T_{l50}$ is constant) and high temperatures ($T_{h50}$ is constant), respectively, and dormancy release shifts temperature range permissive for germination upward and downward, respectively. Type 3 germinates only at intermediate temperatures, and temperature range permissive for germination shifts both downward ($T_{l50}$) and upward ($T_{h50}$) as dormancy is terminated.

Based on the assumption that a population with dormant seeds does not germinate in synchrony, we make a new assumption to mathematically describe synchrony of germination within a population through a simple explanatory parameter. To relax the above assumption, we used the square root of variance or standard deviation (regarded as the distance from the mean of a population) of population thermal parameters describing dormancy and thermal niche. Since seeds have nondeep PD (Type 1, see caption for Figure 2), we used only high‐limit temperatures ($T_{h50}$) to determine synchrony of germination (hereafter SOG), because only this parameter shows changes in dormancy and thermal niche. We estimated SOG using the following equation:

(5) $$\mathrm{SOG}\left(\sigma_{\mathrm{Th}}\right)=\frac{\sum {\left[X_{S}-\mu X_{O}\right]}^{2}}{N}$$

where *σ* is the standard deviation of $T_{h50}$, *X*_S_ and *X*_O_ simulated and observed values (Equation 1), respectively, and *N* population size.

To compute Equation (5), we followed a five‐step method.

1. To find μ in Equation (5), we calculated the mean of the data set, including experimental data showing germination fraction for each percentile and each replicate (from 0.1 to 0.9).
2. Then, we found the deviations (${\left[X_{S}-\mu X_{O}\right]}^{2}$), which show the distance from each data point to the mean.
3. The sum of the deviations (estimated by step 2) was computed ($\sum {\left[X_{S}-\mu X_{O}\right]}^{2}$).
4. The result from Step 3 was divided by the size of the population (*N*), which is the number of data points.
5. Finally, we took the square root of the results from step 4.

### 2.9 Graphical method

To determine whether this species has a delayed germination (risk‐avoidance) strategy, we developed a new graphical method, which included the 20‐year mean temperature at locations to which each population is native. Climate data were obtained through Tutiempo website (http://www.tutiempo.net/en/Climate), and population parameters explaining germination thermal niche were estimated by Equation (1). First, we extracted the mean temperature for each month and year in which the relevant developmental events had taken place. Then, a 20‐year mean temperature was computed. Finally, we plotted the 20‐year mean temperature (from local weather data) against the germination thermal niche constructed for this species in response to local conditions.

### 2.10 Rate of widening of thermal niche during afterripening

To calculate the rate of widening of the thermal niche (hereafter RWTN), which describes changes in thermal range permissive for germination during the storage period (afterripening to overcome dormancy), we modeled the relationship between two variables (storage period at hourly intervals and increases in the high‐temperature limit or thermal niche for germination) by fitting a linear equation to the data. The first variable is an explanatory variable and the second one a dependent variable.

The following equation was used to calculate the rate of widening of thermal niche (RWTN):

(6) $${\text{Storage time}}_{\left(Y\right)}=a+\left(\text{changes in}\;T_{h\;\left[X\right]}\right)\times \left(b\right)$$

where *X* is the explanatory variable, *Y* the dependent variable, *b* the slope of the line and *a* the intercept (the value of ***Y*** when *X* = 0).

To estimate the rate of widening of thermal niche (RWTN), the following equation was derived;

(7) RWTN=1/b

### 2.11 Principal component analysis (PCA)

To examine trait correlations across ecological measurements of populations (see Table 3), principal component analysis (PCA) was performed on germination percentage, descriptive population‐based parameters (lower limit and higher limit temperatures, $T_{l50}$ and $T_{h50}$), synchrony of germination, rate of widening of thermal niche and thermal time required for germination, based on statistical methods implemented within Prism (PCA test was performed using GraphPad Prism version 9.1.2 for Windows, GraphPad Software, www.graphpad.com) and SAS (version 9.3) environments.

### 2.12 Germination timing

To predict germination timing in response to environmental cues and afterripening, we used a nonlinear regression and interpolation function to answer the long‐standing question of when to germinate. The regression line was fitted to long‐term mean temperature and thermal niche quantified by Equation (1). To do so, we predicted germination timing in the field when the mean temperatures at each population intersected the lines showing thermal niche. The nonlinear regression and interpolation function provided predicted thermal values, which are implemented into each panel devoted to populations. Since seeds from the populations are conditionally dormant during development (D) and at maturity (M) and need to afterripen to overcome dormancy and widen thermal niche, the predicted thermal values show the mean temperature that stimulates germination in the fall, suggesting that when temperature reaches the predicted thermal values, germination commences. (There is no overlap between thermal niche and the mean temperatures across these periods.)

## 3 RESULTS

### 3.1 Temperature effects on dormancy

Whereas no or only a moderate fraction of the seeds derived from all nine populations germinated at high temperatures (25°C) during development (two weeks prior to maturity; D = 0%) and even at maturity (M 20%–35%; Figure S5), a moderate‐to‐high proportion completed germination at the cooler temperatures (5 and 10°C; D 20%–80%; M 60%–85%; Figures S1 and S2). Therefore, we investigated the effect of afterripening on germination over a broad temperature range (5 to 25°C). Germination for all populations increased during 1, 2, and 5 months of afterripening. In most populations, the highest germination percentage was observed for seeds afterripened for 2 and 5 months (Figures S1–S3), while some populations (such as Zabol, Tafresh, and Semirom) showed the reserve trend, meaning that even mature seeds (M) germinated to the highest percentage (Figures S1–S3).

### 3.2 Quantification of dormancy

Both immature and mature seeds were dormant and exhibited Type 1 nondeep PD. Changes in dormancy level in response to various periods of afterripening, explained by descriptive parameters in Table 1, resulted in a consistent increase in population‐based parameters describing the dormancy state (widely fluctuating higher temperature limit, $T_{h50}$). Here, we modeled germination behavior of nine populations of *N. sativa* at 5, 10, 15, 20, and 25°C.

**TABLE 1:** Estimated population parameters for fresh and after‐ripened seeds of *Nigella sativa*.

Population	Storage period (month)	Seed status	$T_{l50}$ ^a^	$\sigma_{\mathrm{Tl}}$ ^b^	$T_{h50}$ ^c^	$\sigma_{\mathrm{Th}}$ ^d^	${\mathrm{\theta T}}_{\mathrm{sub}}$ ^e^	$\sigma_{\mathrm{sub}}$ ^f^	${\mathrm{\theta T}}_{\text{supra}}$ ^g^	$\sigma_{\text{supra}}$ ^h^	*R* ^2^ ^i^
Arak	0	D	6.6	7.2	19.3	11.8	29.3	18.3	20	5	.93
	0	M	6.6	4	23	9.31	26.9	7	18	5	.91
	1	AR	6.6	2	26	6.5	26.8	7	17	5	.98
	2	AR	6.6	2	32	4.5	17	6.9	16	5	.98
	5	AR	6.6	2	35	2	15	4.4	16	4	.97
Bajestan	0	D	6.3	4	15	10	26	6	28	4	.93
	0	M	6.3	4	22	7	24	6	28	4	.97
	1	AR	6.3	3	25	6	23	6	25	3	.89
	2	AR	6.3	2	29	5	15	5	20	3	.86
	5	AR	6.3	2	32	2.7	15	5	20	2	.95
Eshkazer	0	D	9	7	17.5	10	30	10	30	20	.92
	0	M	9	6	19.5	8	30	10	30	7	.91
	1	AR	9	6	23.3	5	27	9	20	5	.99
	2	AR	9	3	25.62	3.5	22	6	20	4	.99
	5	AR	9	2.4	30	1.09	22	5	12	4	.82
	0	D	5.8	8	17.5	22	20	8	40	12	.96
Gardmiran	0	M	5.8	6.5	20	14	22	7	20	8	.98
	1	AR	5.8	5	22.5	9.36	15	6	18	4	.92
	2	AR	5.8	4	27	7.5	15	5	19	3	.88
	5	AR	5.8	2.9	30.5	4.8	10	2	15	2	.95
Khaf	0	D	5.5	7	19.5	16	31	7	22	8	.94
	0	M	5.5	5.9	22.9	7.5	30	4	22	8	.99
	1	AR	5.5	4	24	6	25	3	20	8	.87
	2	AR	5.5	3.5	29	3.8	19	2	18	8	.98
	5	AR	5.5	2.5	33	2	10	1.6	15	7	.98
Razan	0	D	4	4	17	11	31	8.4	20	5	.84
	0	M	4	3	23	9.5	23	8	20	5	.89
	1	AR	4	2.8	27	7	26.9	4.4	16	4	.92
	2	AR	4	3	30	3.5	18.8	3.6	16	4	.96
	5	AR	4	3	32	2	17	2	15	4	.95
Semirom	0	D	3	4	22	8	32.6	7.1	20	5	.89
	0	M	3	3	25	7	23	6	16	5	.91
	1	AR	3	3	31.8	5.5	16.6	4	16	4	.88
	2	AR	3	3	34	4	16.1	2	13	4	.96
	5	AR	3	3	35.5	1	12.9	0.8	10	3	.98
Tafresh	0	D	4.8	8	19.6	10	30	8	20	5	.80
	0	M	4.8	3	22	8.5	23.8	7.8	16	4	.89
	1	AR	4.8	3	25	7	18.4	7.2	16	4	.93
	2	AR	4.8	2.5	29	5	15.7	6	15	4	.96
	5	AR	4.8	2.5	33	5	14.9	4	14	4	.96
Zabol	0	D	4.2	3	22	8	29.8	9.1	16	6	.84
	0	M	4.2	1.5	25	6.3	24.3	6.71	15	6	.94
	1	AR	4.2	1.5	28	3.8	19.4	6.2	14	5	.98
	2	AR	4.2	1.5	30	2.5	15.1	4.8	12	5	.95
	5	AR	4.2	1	33	1	13.3	4	12	4	.97

*Note*: The parameters were obtained by simulation of the germination time‐course curves for seeds at the different incubation temperatures (5, 10, 15, 20, and 25°C).

Abbreviations: D, two weeks prior to maturity (immature seeds); M, maturity time; AR, afterripening.

^a^ Mean lower limit temperature for germination of the seed population. The term indicates the lowest temperature that yields 50% germination of the seed population.

^b^ Standard deviation of the values of lower limit temperature for germination ($T_{l50}$) in the seed population.

^c^ Mean higher limit temperature for germination of the seed population. The term indicates the highest temperature that yields 50% germination of the seed population.

^d^ Standard deviation of the values of high‐limit temperature for germination ($T_{h50}$) in the seed population.

^e^ Required amounts of thermal time units (°C days) for completion of germination of 50% of the seed population at the suboptimal temperatures.

^f^ Standard deviation of the thermal time at the suboptimal temperatures.

^g^ Required amounts of thermal time units (°C days) for completion of germination of 50% of the seed population at the supra‐optimal temperatures.

^h^ Standard deviation of the thermal time at the supra‐optimal temperatures.

^i^ Coefficient of determination.

Change in dormancy status varied inconsistently with time and among populations (Table 1). Whereas the Semirom and Razan populations had the narrowest thermal niche (i.e., the lowest $T_{l50}$ of 3 and 4°C, respectively), the Eshkazer, Arak, and Bajestan populations had the highest $T_{l50}$, ranging from 6.3 to 9°C (Table 1). $T_{h50}$ varied considerably among populations and after‐ripening intervals, while $T_{l50}$ remained unchanged, demonstrating that seeds of this species have Type 1 nondeep PD (Table 1, Figure 2). The lowest $T_{h50}$ was for seeds with no afterripening (both D and M), and the $T_{h50}$ was much higher for after‐ripened seeds than for immature and mature seeds, increasing from 15°C to 25°C among populations (Table 1). In spite of these widely different high‐temperature limits among populations, the developed process‐based model fitted germination data well (Table 1).

### 3.3 Synchrony of germination

The pattern of synchrony varied considerably among populations, and asynchrony leveled off with time. Seeds germinated more synchronously within populations with afterripening. Immature and mature seeds of all nine populations exhibited asynchronous germination when they were tested at a gradient of five temperatures from 5 to 25°C in 5°C intervals for all populations (Figure S7, black arrows).Thus, afterripening increased synchrony level among populations until the most synchronous pattern was achieved (Figure S7, green arrows), leading to increased germination. After 5 months of afterripening, degree of synchrony reached its ultimate value (Figure S7, green arrows).

### 3.4 Breadth of thermal niche

**FIGURE 3:**
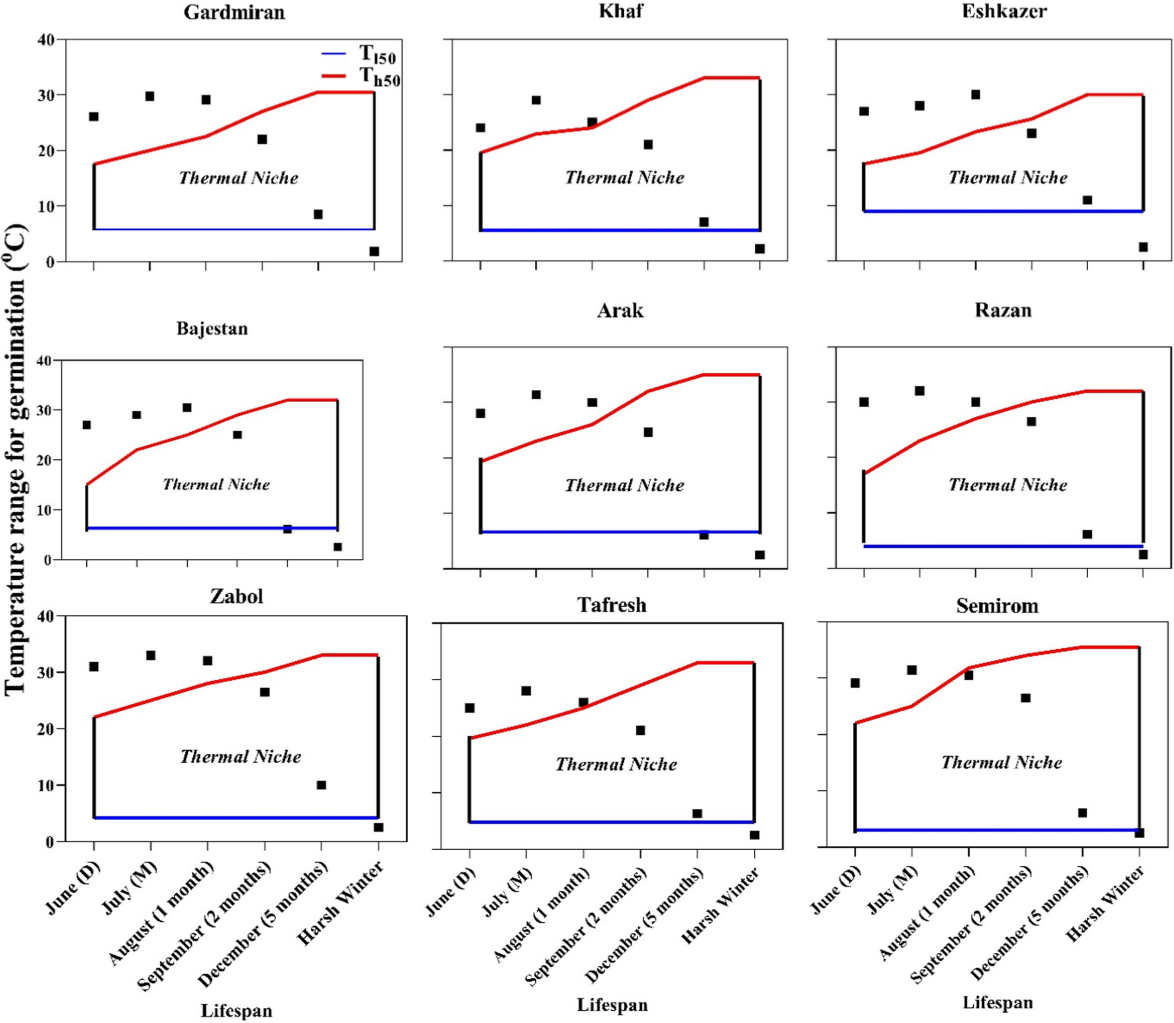
Thermal niche occupied by populations of *Nigella sativa*. Each niche is constructed in response to environmental filtering, particularly temperature. The solid squares represent the mean of 20 temperatures recorded in the location where *N. sativa* is native. A considerable quantity of seeds can germinate whenever mean temperature overlaps with thermal niche. The solid squares outside of the thermal niche mean that environmental cues for germination are not suitable or (may not be perceivable) for this species. $T_{h50}$, mean higher limit temperature; $T_{l50}$, mean lower limit temperature; 1 month, one month of afterripening; 2 months, two months of afterripening; 5 months, five months of afterripening; D, immature seeds; M, mature seeds.

The graphical method showed that each population occupies a given thermal niche (Figure 3, solid squares). Based on the graphical method, germination might occur when the thermal niche overlaps with long‐term thermal condition (solid squares), resulting in the adjustment of germination timing and delay of germination until the overlap occurs. Afterripening had a considerable impact on widening the thermal niche: the more dormancy that was released the wider the thermal niche.

### 3.5 Rate of widening the thermal niche during dry afterripening

Rate of widening the thermal niche differed significantly among some populations (Table 2). The Zabol and Razan populations had the highest rate of widening of the thermal niche, whereas changes in the thermal niche during afterripening were not significant in Razan (Table 2; *p* = .0937). Thus, germination of Razan population became synchronous more slowly than it did for seeds from other populations with a low rate of widening of the thermal niche, in which afterripening enhanced synchrony values rapidly, namely for Arak and Tafresh.

**TABLE 2:** Estimated parameters for the linear model (Equation 6) fitted to observed data to calculate the rate of widening of thermal niche at hourly intervals during afterripening.

Population	*a* (intercept)	*b* (slope of the line)	RWTN^a^ (h per 1°C → Th)	*p* Value	*R* ^2^
Arak	24.29	0.003274 ± 0.0009173	305.5^ns^	.0703	.86
Bajestan	23.14	0.002679 ± 0.0006391	373.3^ns^	.0525	.89
Eshkazer	20.65	0.002748 ± 0.0004617	363.9**	.0271	.94
Gardmiran	20.86	0.002877 ± 0.0006115	347.6**	.0423	.91
Khaf	23.05	0.002897 ± 0.0005693	345.2**	.0365	.92
Razan	24.71	0.002282 ± 0.0007523	438.3^ns^	.0937	.82
Semirom	23.49	0.002450 ± 0.001203	408.1^ns^	.1785	.81
Tafresh	22.96	0.002976 ± 0.0005546	336.0**	.0330	.93
Zabol	26.00	0.002083 ± 0.0004150	480.0**	.0375	.92

*Note*: **Significant, ^ns^ nonsignificant.

^a^ Rate of widening thermal niche during dry afterripening. *F*‐test was used as a procedure to assess test of significance.

### 3.6 Principal component analysis (PCA)

**FIGURE 4:**
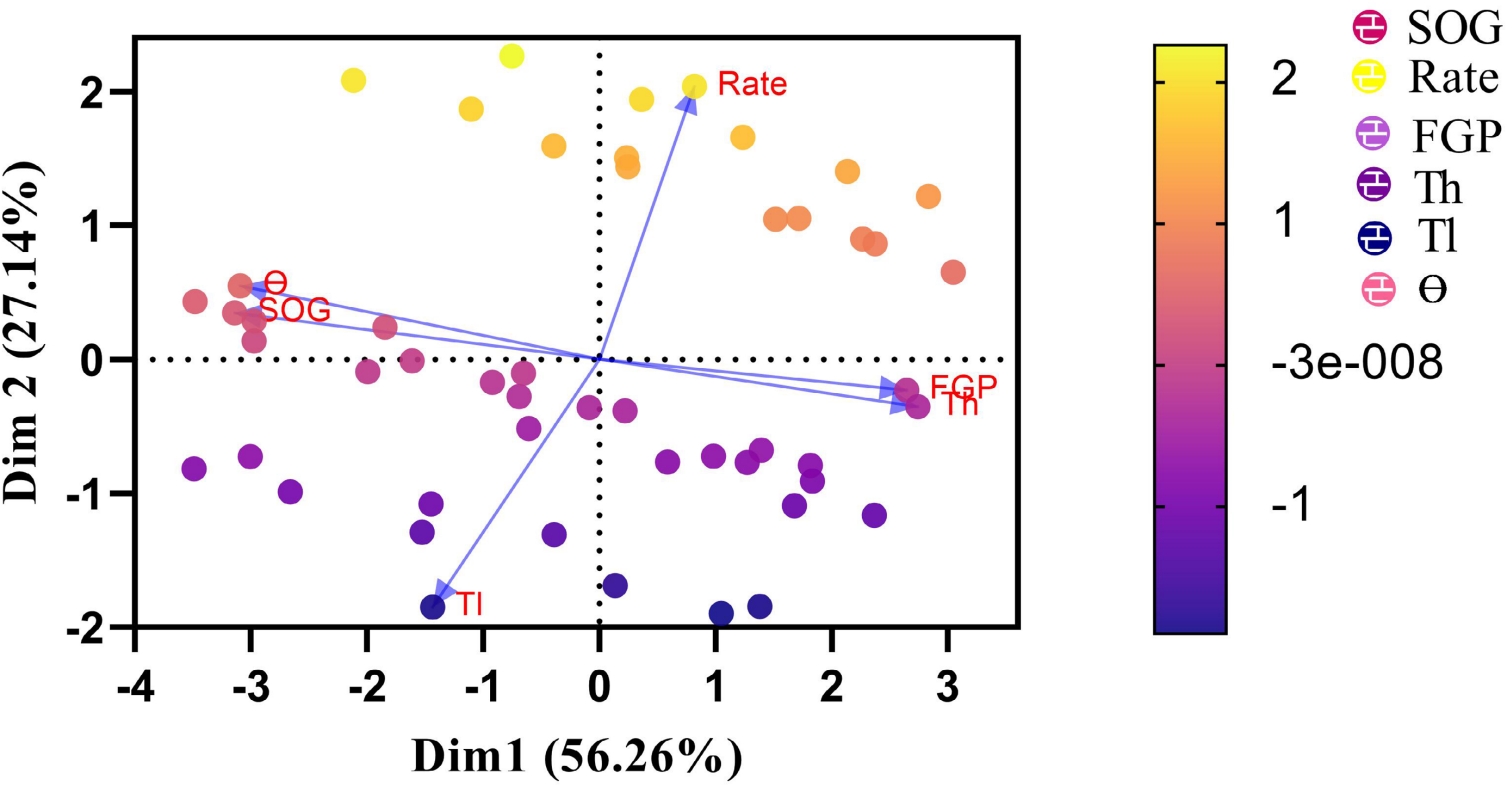
Principal component analysis results of ecological measurements for nine populations of *Nigella sativa*. Data plotted describe the first two principal components, which explain the majority of variance. $T_{h50}$, higher temperature limit; $T_{l50}$, lower temperature limit; FGP, final germination percentage; Ɵ, thermal time required for germination; Rate, rate of widening of thermal niche; SOG, synchrony of germination.

Using PCA, populations and ecological measurements fell into different categories (Figure 4; Table 3). The first two components described 56.26% of the variance (Figure 4). The analysis showed that dormancy pattern can change during dry afterripening and that the changes differ among populations (Figure 4). Thermal time requirements and synchrony of germination explained 1.20% and 2.70% of the variance among ecological measurements, which had the least variance among measurements. Of the ecological measurements, final germination percentage (FGP) and $T_{h50}$ were highly correlated with each other, while $T_{l50}$ and rate of widening of thermal niche showed no correlation with other parameters. Thermal time required for germination and synchrony of germination were highly correlated.

**TABLE 3:** Principal component analysis of dormancy and germination for different germination parameters.

Variable	PCA correlations	Proportion of variance (%)
FGP^a^	0.926041	56.26
Th^b^	0.959628	23.48
Tl^c^	−0.38151	10.89
Rate^d^	0.285922	5.47
SOG^e^	−0.83397	2.70
Ɵ^f^	−0.82117	1.20

^a^ Final germination percentage.

^b^ Higher temperature limit.

^c^ Lower temperature limit.

^d^ Rate of widening of thermal niche.

^e^ Synchrony of germination.

^f^ Thermal time required for germination.

Since the first principal component captured 52.26% of the variance, species with higher germination capacity are better able to come out of dormancy and take advantage of dormancy status to synchronize germination. The second principal component described 23.48% of the variance shows that higher temperature limit (in this case, the parameter showing the widening of thermal niche), which was affected by dormancy level, directly contribute to germination timing and delayed germination strategy, leading to divergence in germination behavior across populations (Table 3).

### 3.7 Prediction of germination timing

Germination of all populations occurred in a window of time ranging from 2 to 5 months of afterripeing. Predicted thermal values for temperature that cues germination in the field varied considerably among populations, depending on the environmental conditions and thermal history they have experienced. The Semirom, Zabol, and Khaf populations showed the highest predicted thermal values of 29.55, 28, and 27.62°C, respectively (Figure S8), and the Eshkazar and Gardmiran populations showed the lowest predicted values of 21.10 and 24.12°C, respectively (Figure S8).

## 4 DISCUSSION

Sensitivity to temperature is a fundamental ecological phenomenon for plants growing in unpredictable habitats where successful germination is highly dependent on the spatiotemporal variability in temperature. Temperature may play a primary regulatory role in determining the germination thermal niche by modulating seed dormancy and subsequently the thermal niche and by determining synchrony (Batlla & Benech‐Arnold, 2015; Soltani, Baskin, et al., 2017; Soltani, Gruber, et al., 2017). A study by Alshammari (2017) found that seeds of *N. sativa* are responsive to temperature with an optimum of 23°C and that germination did not occur below 15°C or above 25°C. Changes in germination patterns as seeds afterripen (Figure 3) are consistent with an increase in population‐based parameters describing the dormancy state (widely differing higher temperature limit, $T_{h50}$). We found changing patterns of seed responses to temperature among populations of *N. sativa*, with marked variation in germination at high temperatures (Figures S3–S5). Furthermore, delayed germination strategy was associated with long‐term environmental conditions were obvious. The results show that dormancy patterns might be relevant to environmental conditions at each local site rather than at the landscape scale, suggesting that differential germination response to the environment of each population site underlies environmental regulation of seed dormancy (Cavallaro et al., 2016; Krichen et al., 2014; Rotundo et al., 2015; Tognetti et al., 2019).

Conditional dormancy is a transitory state between dormancy and nondormancy in which seeds are only able to germinate within a narrow temperature range (limited thermal niche), and as seeds become nondormant, they progressively gain the ability to germinate in a broader temperature range until the widest thermal niche for germination is reached (Baskin & Baskin, 2014; Maleki et al., 2021). In our study populations of *N. sativa* with conditional dormancy had different germination strategies via different germination niches with variable breadth and different synchrony patterns, which is a potential adaptive trait underlying adaptive strategies and creates a dynamic status change in response to dormancy level. Conditional dormancy is a strategic mechanism synchronizing germination with favorable conditions by creating a dynamic status altering the breadth of germination thermal niche in response to seasonal cues (Baskin & Baskin, 2014; Maleki et al., 2021; Soltani et al., 2022). Maleki et al. (2021) showed that conditional dormancy adjusted germination timing of *Brassica napus* in response to environmental cues. This suggests that conditional dormancy enables a species to delay germination of freshly matured seeds coincident with onset of harsh and dry summer conditions, and then it optimizes germination by synchronizing germination with early autumn in which suitable conditions for germination are expected (Figure 3).

In addition to sensing unfavorable conditions, there is an underlying mechanism that drives conditional dormancy. In *B. napus*, for example, thermal after‐ripening time, viewed as thermal units that must be accumulated during summer to allow seeds to start germinating, helps this species track environmental conditions (Maleki et al., 2021). In explaining the previous concept in the an underlying mechanism that drives conditional dormancy, several issues must be taken into account. (1) For species requiring a certain period of dormancy‐breaking treatment (either stratification or afterripening), a given amount of thermal units is needed, providing a regulatory mechanism that delays germination until the thermal units requirements for germination are fulfilled (Figure S6). (2) This sensory mechanism implies that the need for given dormancy‐breaking treatments may have an evolutionary underpinning. Thus, the requirements for germination mean that seeds germinate at the onset of favorable conditions for seedling establishment (Figure 3). In agreement with our hypothesis about underlying mechanisms that drive conditional dormancy, Carruggio et al. (2021) concluded that conditional dormancy can serve as a highly efficient adaptation strategy that allows species with type 1 nondeep PD to avoid stressful conditions in areas with an unpredictable Mediterranean climate.

Interestingly, for after‐ripened seeds (relative to immature and mature seeds), all populations had higher and more synchronous germination, indicating that dormancy had changed and that the seed population had become less dormant. Seeds are produced in early summer and afterripen during the remainder of the summer; thus, immature, mature, and 1‐month after‐ripened samples coincide with the summer season (Figure 1). We conclude that thermal time experienced during summer may play a part as an underlying mechanism that drives conditional dormancy, meaning that being responsive to afterripening to overcome seed dormancy may have evolutionary underpinnings.

Within‐population variation in germination behavior also may indicate differences in germination strategies. For example, the Semirom and Arak populations, which occupy the driest sites, had the highest $T_{h50}$ among the nine populations, suggesting that they may be adapted to the conditions in the early growing season (autumn). Conversely, the lower $T_{h50}$ in the other seven populations may indicate a more conservative germination strategy, that is, seeds germinate only in a narrower thermal niche. Maximum germination capacity and the rate of widening of the thermal and germination niches in after‐ripened seeds were significantly correlated with long‐term mean temperature (Figure 3, Table 3). The simulated thermal niche matched well with the observed germination niche. Some populations were characterized by high θT values, with extremes of 32.6 and 31°C for Semirom and Razan, respectively. This is of ecological significance, since these populations may delay germination because of a higher thermal requirement than populations with a lower thermal requirement, such as Gardmiran (Table 1). Thus, populations with a low rate of widening of the thermal niche might respond better to afterripening (accumulated thermal time) than those with a high rate of widening of the thermal niche (Table 2, Figure S6).

Some generalizations about the descriptive population‐based parameter ($T_{h50}$) can be made for each population (Table 1). There was a consistent trend between estimated values for $T_{h50}$ and θT, primarily due to dormancy, since the more dormancy decreased the less thermal time was required for germination (Figure S6). Another ecological aspect demonstrated here is that the magnitude of $T_{h50}$‐thermal after‐ripening time slope varied with storage period. $T_{h50}$ increased among populations as thermal after‐ripening time increased, with slopes ranging from 5.53 (Khaf) to 5.47 (Arak) (Figure S6).

Germination synchrony varied considerably among populations. Immature and mature seeds of all populations showed signs of asynchronous germination at the prevailing temperatures (a gradient of five temperatures from 5 to 25°C in 5°C intervals for all populations) (Figure S7, black arrows). Afterripening released seed dormancy, and consequently germination timing was adjusted by delaying germination, thereby avoiding drought in summer. Thus, the more dormancy that was released the more synchronous germination became (Figure S7, green arrows).

Studies using dynamic models have indicated that risk‐spreading strategies are beneficial and that species may take advantage of integrated strategies that incorporate predictive plasticity of phenology and timing with bet‐hedging (Gremer et al., 2016). The need for afterripening delayed germination based on long‐term climate data, the loss of dormancy with afterripening and delay of germination until late autumn (December).

Population‐based parameters are useful tools for quantifying germination strategies and germination timing (Donohue et al., 2015). For example, higher germination plasticity (wider thermal niche) can be obtained when the descriptive parameter ($T_{h50}$) has a higher value. Threshold‐type responses to temperatures (i.e., lower or higher limit temperatures) have been widely used to quantitatively describe variation in germination and dormancy loss for several species in the Brassicaceae (Del Monte & Tarquis, 1997; Maleki et al., 2021; Soltani, Baskin, et al., 2017) and of *Polygonum aviculare* (Polygonaceae) (Batlla & Benech‐Arnold, 2015). Ecologically meaningful parameters (i.e., $T_{l50}$ and $T_{h50}$) are powerful tools for quantification of degree of seed dormancy and identifying types of PD. In Type 1 nondeep PD, $T_{l50}$ remains unchanged and $T_{h50}$ increases progressively and simultaneously. Soltani, Baskin, et al. (2017) found that cardinal temperatures for final germination percentage can change during dormancy breaking within a population. They explained that seeds with types 1, 2, and 3 of nondeep PD have a continuum of transition stages known as conditional (or relative) dormancy but that those seeds with types 4, 5, and 6 do not. Maleki et al. (2021) demonstrated that changes in population‐based parameters can be considered as degree of dormancy.

Based on the graphical method and estimated parameters (Table 1) developed here, we showed that thermal niche varied considerably among populations. Thus, each population may have evolved a specific strategy in response to the environment of the local site (Figure 3; Table 1). The graphical method showed that the germination niche might be different when thermal niche overlaps with long‐term thermal conditions (Figure 3, solid squares). Consequently, dormancy can delay germination until the temperature overlap occurs, leading to adaptive delayed germination that can increase survival by synchronizing germination with favorable conditions. Breadth of the thermal niche differed among populations. The 20‐year mean temperature for the Semirom and Zabol populations, which had the widest thermal niches, was higher than that of the other population sites, suggesting that plants experiencing higher temperatures may have a broader thermal niche (Figure 3; Table 1).

Species with a Mediterranean origin (such as *N. sativa*) have evolved a germination niche for low temperatures in autumn. The highest germination percentage was at 5–15°C, which coincides with autumn, suggesting an adaptation that avoids summer drought (e.g., De Vitis et al., 2014; Fenner & Thompson, 2005; Ne'eman & Goubitz, 2000). Germination of *N. sativa* seeds at low temperatures (5–15°C) in autumn agrees with the germination requirements of other Mediterranean species (Ne'eman & Goubitz, 2000; Fenner & Thompson, 2005; De Vitis et al., 2014). Moreover, the role of differentiation in the germination niche and diurnally fluctuating temperatures in germination strategies of species with a Mediterranean origin is well‐documented (Saatkamp et al., 2011).

Germination timing can explain not only the fate of newly emerged seedlings but also the environment in later life stages (Gremer et al., 2020). As such, selection on morphological traits, life history timing, germination niche and population persistence and regulation may be affected by germination timing. Our results illustrate that kind of dormancy and an after‐ripening requirement for germination have the potential to drive the timing and capacity for germination. Thus, responsiveness of seeds to environmental temperature gradients drive the evolution of their niche. Differences in germination patterns among populations may be mediated by population responsiveness to afterripening and accumulated thermal time. Studies also have indicated that autumn‐germinating plants with high maximum temperature for germination can start germinating in early autumn, leading to more opportunities for germination (opportunistic) and thus higher germination percentages (Huang et al., 2016; Soltani, Baskin, et al., 2017; Soltani, Gruber, et al., 2017). Germination capacity and high cumulative germination fractions are characteristics of the germination niche of different populations with practical implications for bet‐hedging (Gremer & Venable, 2014).

The various germination patterns among populations of *N. sativa* showed multiple ways in which this species delays germination and synchronizes it with a suitable growing season. Our results reveal that loss of dormancy via afterripening and passage through conditional dormancy are of significance in the timing of germination and in seedling survival. Thus, *N. sativa* delays germination via conditional dormancy with a narrow breadth of the germination niche at dispersal, thereby avoiding summer mortality until the thermal requirement for germination is fulfilled during summer. Gremer et al. (2020) showed that the optimal germination date can impact seedling survival, fruit production, germination patterns, and later developmental transitions. Plants from seeds that germinate at maturity (early summer for *N. sativa*) may have the opportunity to capitalize on resources such as light availability. However, unpredictable environmental conditions such as lack of rainfall that could lead to seedling mortality early in the season thereby negating any advantage gained from early germination (Baskin & Baskin, 2014; Donohue et al., 2010; Gremer et al., 2020).

We identified a clear difference in the thermal niche across populations of *N. sativa*, which allows each of them to germinate under current conditions. More interestingly, the breadth of the thermal niche that has evolved in the nine populations corresponds with the 20‐year mean temperature. Populations with a broad thermal niche for germination may have wide environmental tolerance and are likely to be affected less by environmental unpredictability (Finch et al., 2019).

## 5 CONCLUDING THOUGHTS

Degree of dormancy underlies the breadth of the thermal niche of *N. sativa*, and afterripening had incremental effects on dormancy loss that resulted in a broader thermal niche. Using long‐term climatic data, we showed that *N. sativa* has a delayed germination strategy that avoids summer mortality and a dormancy pattern that synchronizes germination with the appropriate growing season in autumn. The population‐based model employed in our study precisely described the germination strategy of *N. sativa* and provided a quantitative framework for understanding germination behavior and thermal niche across different environments. Breadth of the thermal niche was narrow at seed maturity, which prevented germination and thus seedling mortality due to drought. The Semirom and Arak populations had the highest $T_{h50}$ among the nine populations of *N. sativa*, suggesting that they are the ones best adapted to environmental conditions in the early growing season. On the contrary, the lower $T_{h50}$ in the other seven populations may indicate a more conservative germination strategy.

## AUTHOR CONTRIBUTIONS

**Keyvan Maleki:** Data curation (equal); formal analysis (equal); software (equal); writing – original draft (equal). **Carol C. Baskin:** Conceptualization (equal); validation (equal); writing – review and editing (equal). **Jerry M. Baskin:** Conceptualization (equal); validation (equal); writing – review and editing (equal). **Mohadeseh Kiani:** Investigation (equal); software (equal); visualization (equal). **Iraj Alahdadi:** Investigation (equal); software (equal); visualization (equal). **Elias Soltani:** Conceptualization (equal); supervision (equal); validation (equal); writing – review and editing (equal).

## CONFLICT OF INTEREST

The authors declare no conflict of interest.

## Supporting information


Appendix S1

## Data Availability

The supplementary materials have been deposited to Dryad. The materials include raw data, the supplementary material and a readme file. The file can be found at https://doi.org/10.5061/dryad.9zw3r22h1.
