# Supplementary material for: Seed germination thermal niche differs among nine populations of an annual plant: A modeling approach
Source: Ecol Evol. 2022 Aug 26;12(8):e9240. doi: 10.1002/ece3.9240 (PMC9412254; doi:10.1002/ece3.9240)
Supplement: Supplementary file 1 — Appendix S1 [file ECE3-12-e9240-s001.docx]

**Seed germination thermal niche differs among nine populations of an annual plant: a modelling approach**

**Description of the conceptual approach used for the quantification of dormancy and germination parameters of nine populations of *Nigella sativa***

To quantitatively describe changes in the thermal range permissive for seed germination and thus width of the thermal niche during dormancy release and after-ripening, we used a mathematical model based on the conceptual model presented in Batlla and Benech-Arnold (2015). Here, we developed a conceptual framework for interpretation of the breadth of thermal niche of focal populations of *Nigella sativa*. Briefly, the model assumes that the thermal range permissive for germination is limited by two thresholds temperatures ($T_{h50}$ and $T_{l50}$_,_ the higher and lower limit temperatures for seed germination, respectively). Both parameters are normally distributed within the seed population characterized by mean values of $T_{h50}$ and $T_{l50}$ and their corresponding explanatory parameters (standard deviations, *σ*_Th_ and *σ*_Tl_). As dormancy decreases during after-ripening, the temperature range permissive for germination and thus the thermal niche gradually widens until it is maximal (basically by changes in $T_{h50}$, for *N. sativa*), while as dormancy is expressed (*D, M) the range of temperatures under which germination occurs narrows until it is no longer possible at any temperature (full dormancy is reached). Once the prevailing temperature is within the thermal range for seed germination of a certain seed fraction, the germination rate of that fraction is predicted based on the accumulation of thermal time above the base temperature (T_b_) for the sub-optimal thermal range or below the maximal temperature (T_m_) for the supra-optimal thermal range.

**Experiments**

Data from each experiment were divided into five parts: evaluation of primary dormancy of immature and mature seeds and analysis of three after-ripening periods.

a. Degree of primary dormancy 2 weeks prior to maturity and at harvest time

Using data for nine populations, thermal parameters that characterize degree of primary dormancy and germination dynamics were estimated (Table 1). These parameters include the following.

1. Parameters related to degree of dormancy.
   1. $T_{l50}$: the lowest temperature at which seeds can germinate.
   2. $T_{h50}$: the highest temperature at which seeds can germinate.
   3. θ_Tl_ and θ_Th_: standard deviations of $T_{l50}$ and $T_{h50}$_,_ respectively.
2. Parameters associated with germination dynamics.
   1. At the sub-optimal range: Base temperature (T_b_) and optimal temperature (T_o_) for germination were 0 and 5-20 °C, respectively. Thermal time (TT) and its standard deviation.

To estimate cardinal temperatures, we used the following dent-like equation:

*R = ((T - T_b_) / (T_o1_ - T_b_)) × Rmax if T_b_ < T < T_o1_*

*R = ((T_c_ - T) / (T_c_ - T_o2_)) × Rmax if T_o2_ < T < T_c_*

*R = Rmax if T_o1_ ≤ T ≤ T_o2_*

*R = 0 if T ≤ T_b_ or T ≥ T*

The fit between observed and predicted data was evaluated using the coefficient of determination (R2) and root-mean-square error (RMSE):

R2=1-[∑(y_obs_-y_pred_)^2^/∑(y_obs_-ȳ_pred_)^2^]

RMSE=$\sqrt{\frac{\sum_{i=0 \left( pi-0i \right)}^{n} 2}{N-1}}$

- 1. At the supra-optimal range: a maximum temperature (Tm) of 30 °C was estimated for germination. Thermal time (TT) and the standard deviation of Tm.

**Germination dynamics after undergoing after-ripening:**

Seed stored at 20 °C showed changes in thermal parameters that characterizes the degree of dormancy. After-ripening was associated with (i) an increase in germination percentages (and rates), (ii) an increase in $T_{h50}$ and (iii) no change in $T_{l50}$, which remained constant. In general, changes in $T_{h50}$ differed inconsistently among populations. However, differences among seed batches were detected.

**Table S1.** Information on the nine study populations.

| **Population** | **Location** | **MAT^a^(°C)** | **MAP^b^(mm)** | **Climate classification^c^** | **Elevation (m)** |
| --- | --- | --- | --- | --- | --- |
| Arak | 34°00'N 49°40'E | 14.6 | 375 | Subtropical (Mediterranean) | 1750 |
| Bajestan | 34.52°N58.17°E | 23.0 | 207 | Arid and Semi-arid | 1234 |
| Eshkazer | 32.02°N53.47°E | 20.4 | 66 | Subtropical Desert | 1216 |
| Gardmiran | 35.09° N 47.36° E | 15.0 | 414. | Humid Continental Climate | 200 |
| Khaf | 34.57°N 58.82°E | 15.2 | 353 | Arid and Semi-arid | 995 |
| Razan | 35°23'N49°02'E | 12.9 | 408 | Cold semi-arid Climate | 2630 |
| Semirom | 31.41°N51.56°E | 15.8 | 129 | Cold and Temperate | 2000 |
| Tafresh | 34.69°'N 51.01°'E | 23.0 | 226 | Subtropical Steppe | 1912 |
| Zabol | 31°00'N 61°32'E | 22.0 | 240 | Subtropical Desert | 483 |

^a^ Mean annual temperature; ^b^ Mean annual percipitation; ^c^according to to the de Martonne climate classification (Raziei *et al.,* 2017)

**Table S2.** Estimated cardinal temperatures for non-dormant seeds of each population.

| **Population** | **Tb(°C)^a^** | **To1(°C)^b^** | **To2(°C)^c^** | **Tm(°C)^d^** | **R2^e^** | **Rmse^f^** |
| --- | --- | --- | --- | --- | --- | --- |
| Arak | 0 | 6.78 | 18.36 | 30 | 0.99 | 0.00052 |
| Bajestan | 0 | 6.52 | 19.56 | 30 | 0.99 | 0.00037 |
| Eshkazer | 0 | 4.78 | 18.23 | 30 | 0.98 | 0.00047 |
| Gardmiran | 0 | 7.22 | 19.34 | 29.65 | 0.92 | 0.00062 |
| Khaf | 0 | 5.63 | 20.45 | 28.74 | 0.99 | 0.00045 |
| Razan | 0 | 5.96 | 20.12 | 30 | 0.99 | 0.00078 |
| Semirom | 0 | 5.00 | 19.45 | 30 | 0.98 | 0.00049 |
| Tafresh | 0 | 5.41 | 20.65 | 30 | 0.98 | 0.00076 |
| Zabol | 0 | 6.23 | 20.74 | 30 | 0.98 | 0.000 |

a, base temperature; b, lower optimum temperature; c, upper optimum temperature; d, maximum temperature; e, coefficient of determination representing the proportion of the variation in the dependent variable; f, root mean square error showing the standard deviation of the residuals.


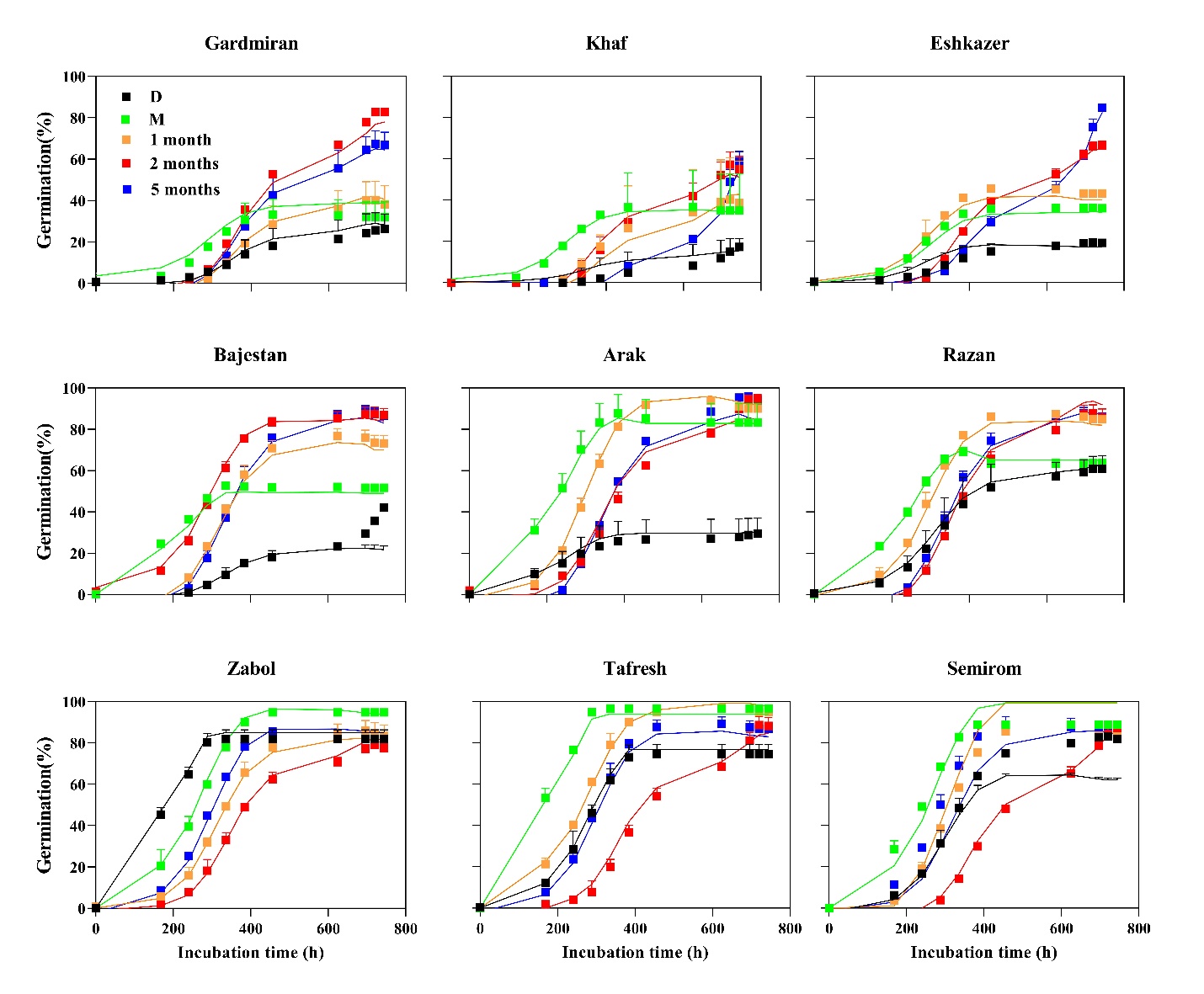


**Figure S1.** Observed (symbols) and simulated (lines; equation 1) germination (%) for seeds of the nine populations of *Nigella sativa* incubated at 5 ^o^C. Treatments are indicated inside first panel. Symbols represent the mean (n= 3). M, maturity; D, development. 1 month, one month of afterripening; 2 months, two months of afterripening; 5 months, five months of afterripening. Three replicates of 20 seeds for each treatment were used. Error bars show standard error.


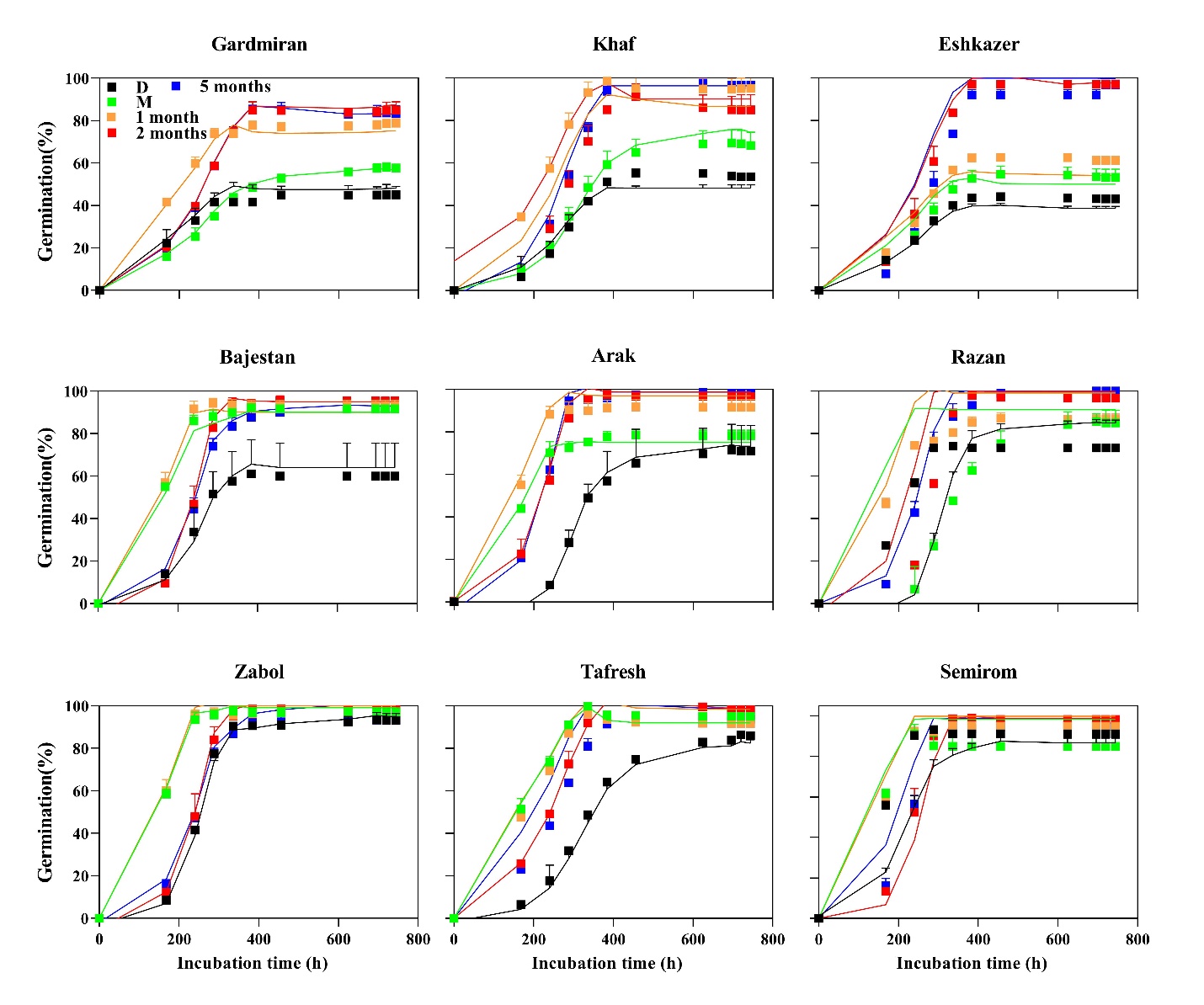


**Figure S2.** Observed (symbols) and simulated (lines; equation 1) germination (%) for seeds of the nine populations of *Nigella sativa* incubated at 10 ^o^C. Treatments are indicated inside first panel. Symbols represent the mean (n= 3). M, maturity; D, development. 1 month, one month of afterripening; 2 months, two months of afterripening; 5 months, five months of afterripening. Three replicates of 20 seeds for each treatment were used. Error bars show standard error.


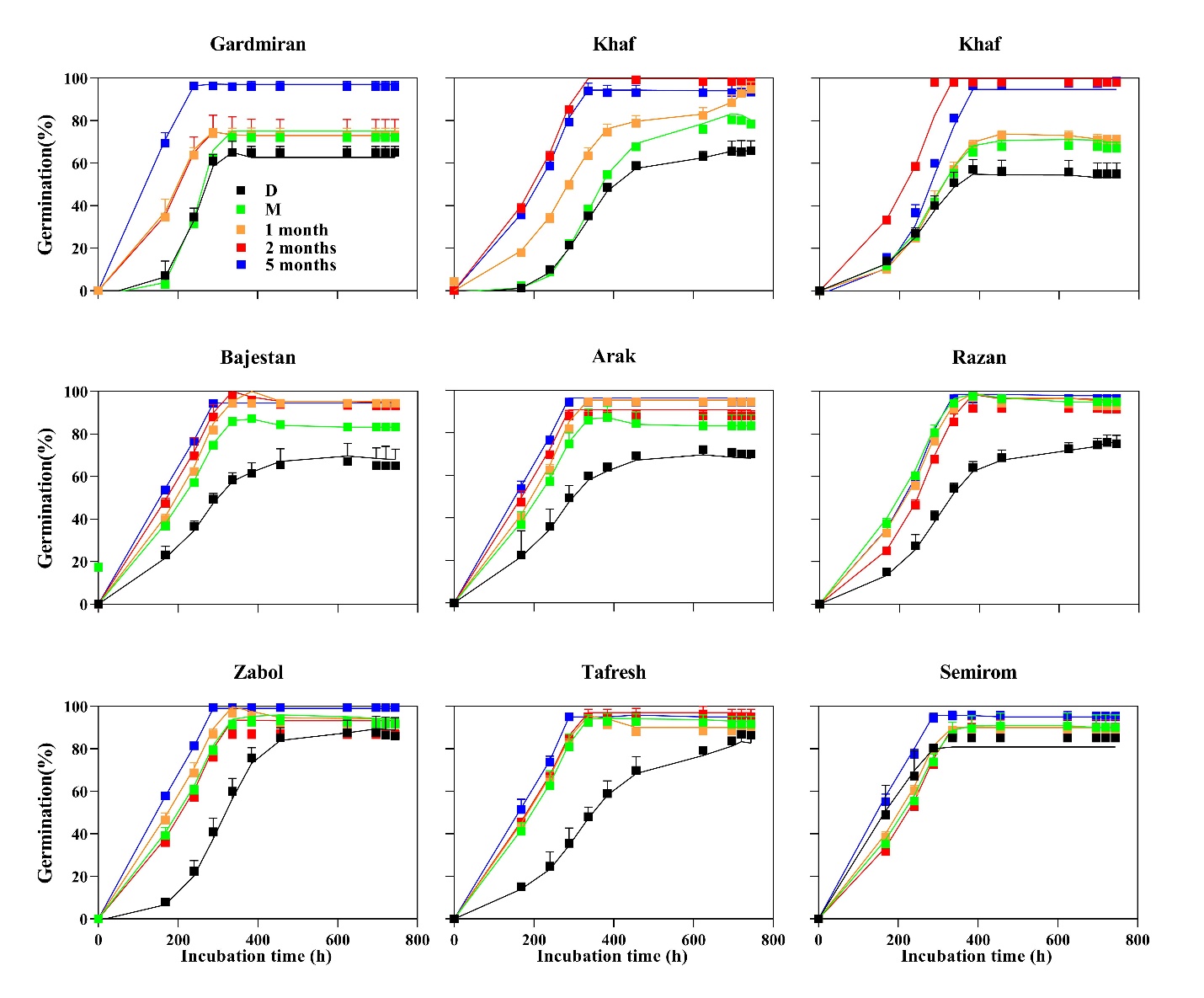


**Figure S3.** Observed (symbols) and simulated (lines; equation 1) germination (%) for seeds of the nine populations of *Nigella sativa* incubated at 15 ^o^C. Treatments are indicated inside first panel. Symbols represent the mean (n= 3). M, maturity; D, development. 1 month, one month of afterripening; 2 months, two months of afterripening; 5 months, five months of afterripening. Three replicates of 20 seeds for each treatment were used. Error bars show standard error.


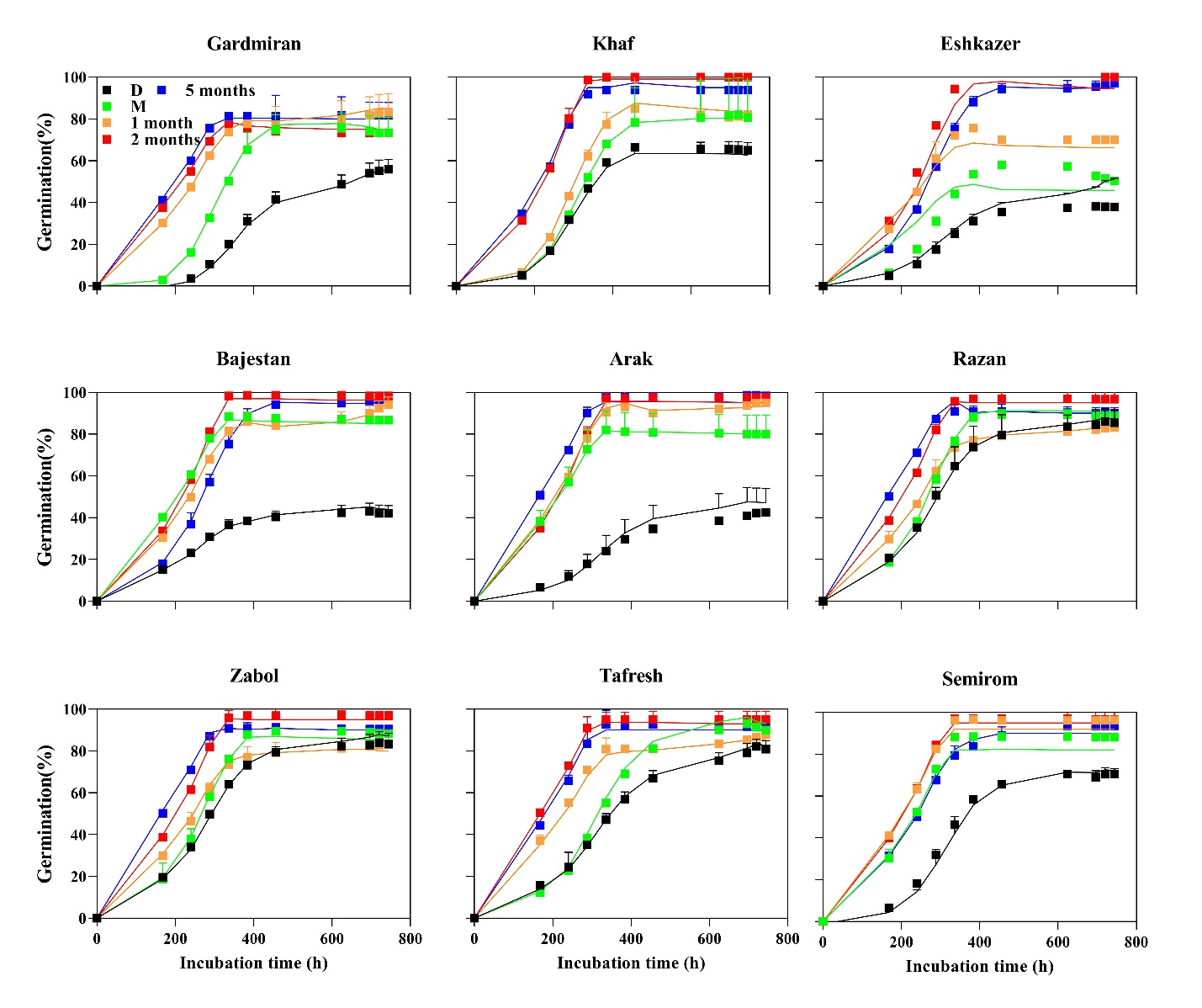


**Figure S4.** Observed (symbols) and simulated (lines; equation 1) germination (%) for seeds of the nine populations of *Nigella sativa* incubated at 20 ^o^C. Treatments are indicated inside first panel. Symbols represent the mean (n= 3). M, maturity; D, development. 1 month, one month of afterripening; 2 months, two months of afterripening; 5 months, five months of afterripening. Three replicates of 20 seeds for each treatment were used. Error bars show standard error.


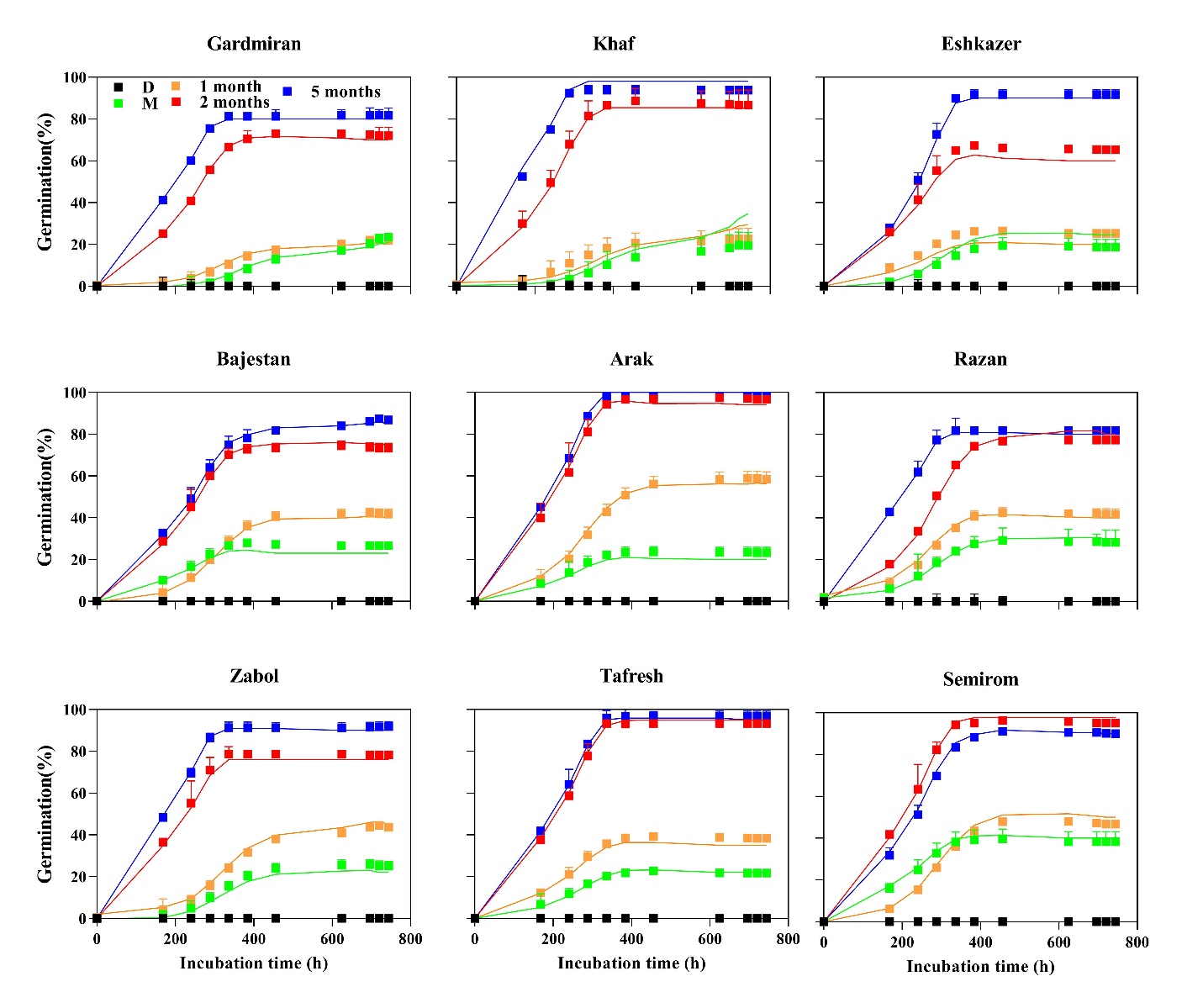


**Figure S5.** Observed (symbols) and simulated (lines; equation 1) germination (%) for seeds of the nine populations of *Nigella sativa* incubated at 25 ^o^C. Treatments are indicated inside first panel. Symbols represent the mean (n= 3). M, maturity; D, development. 1 month, one month of afterripening; 2 months, two months of afterripening; 5 months, five months of afterripening. Three replicates of 20 seeds for each treatment were used. Error bars show standard error.


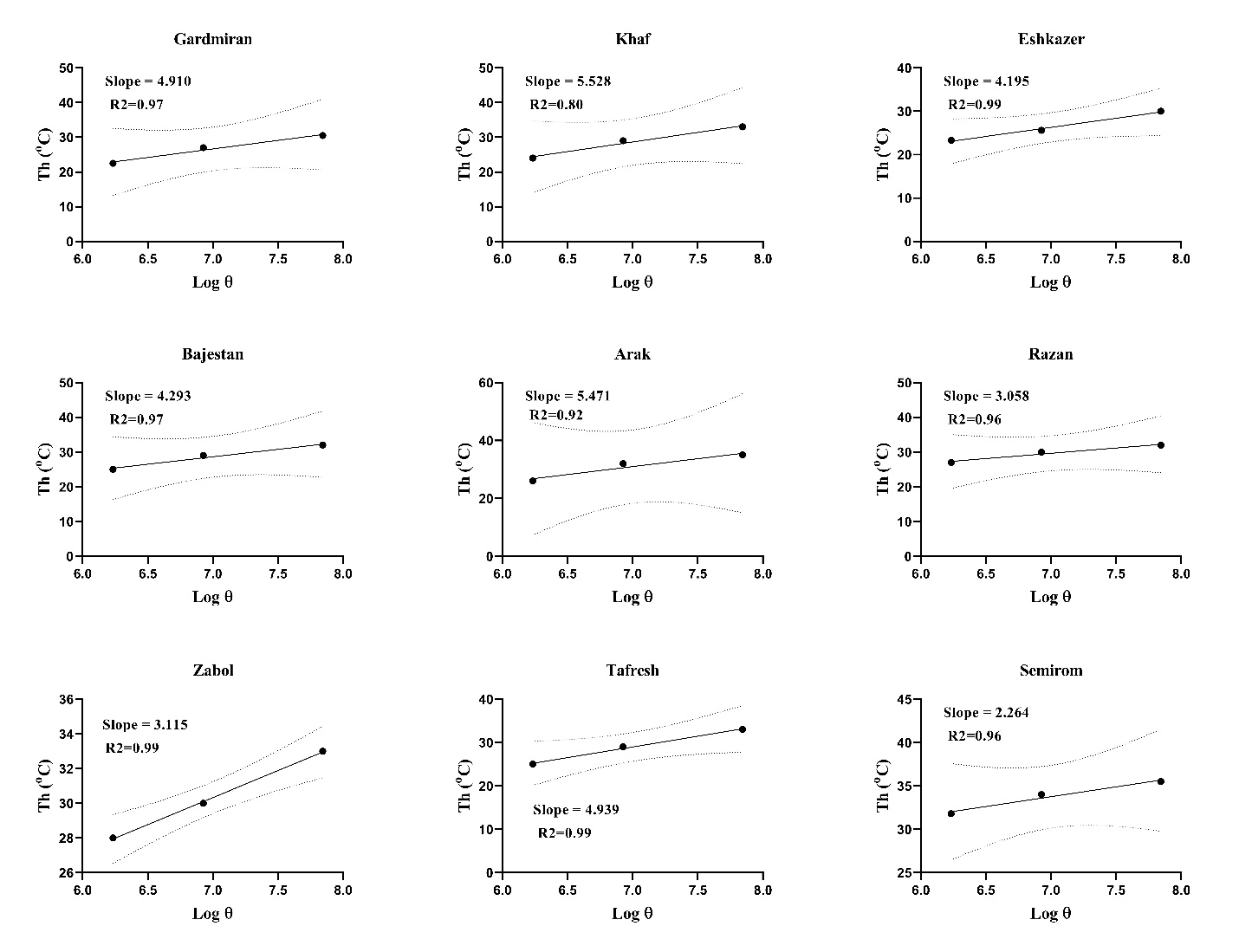


**Figure S6.** Estimated values of the thermal niche parameter (Th, equation 1) for different populations of *Nigella sativa* plotted against thermal after-ripening time (estimated by $\theta AT=\left( Ts-Tsl \right)tar$ proposed by Allen et al., 2007) for dormancy loss and the subsequent widening of thermal range permissive for germination. The fitted lines correspond to the linear regression model. The dotted lines represent 95 % confidence interval. Log-transformation was applied to normalize the data. Th shows mean higher limit temperature. Ɵ indicates thermal time required to reach 50 % of germination.


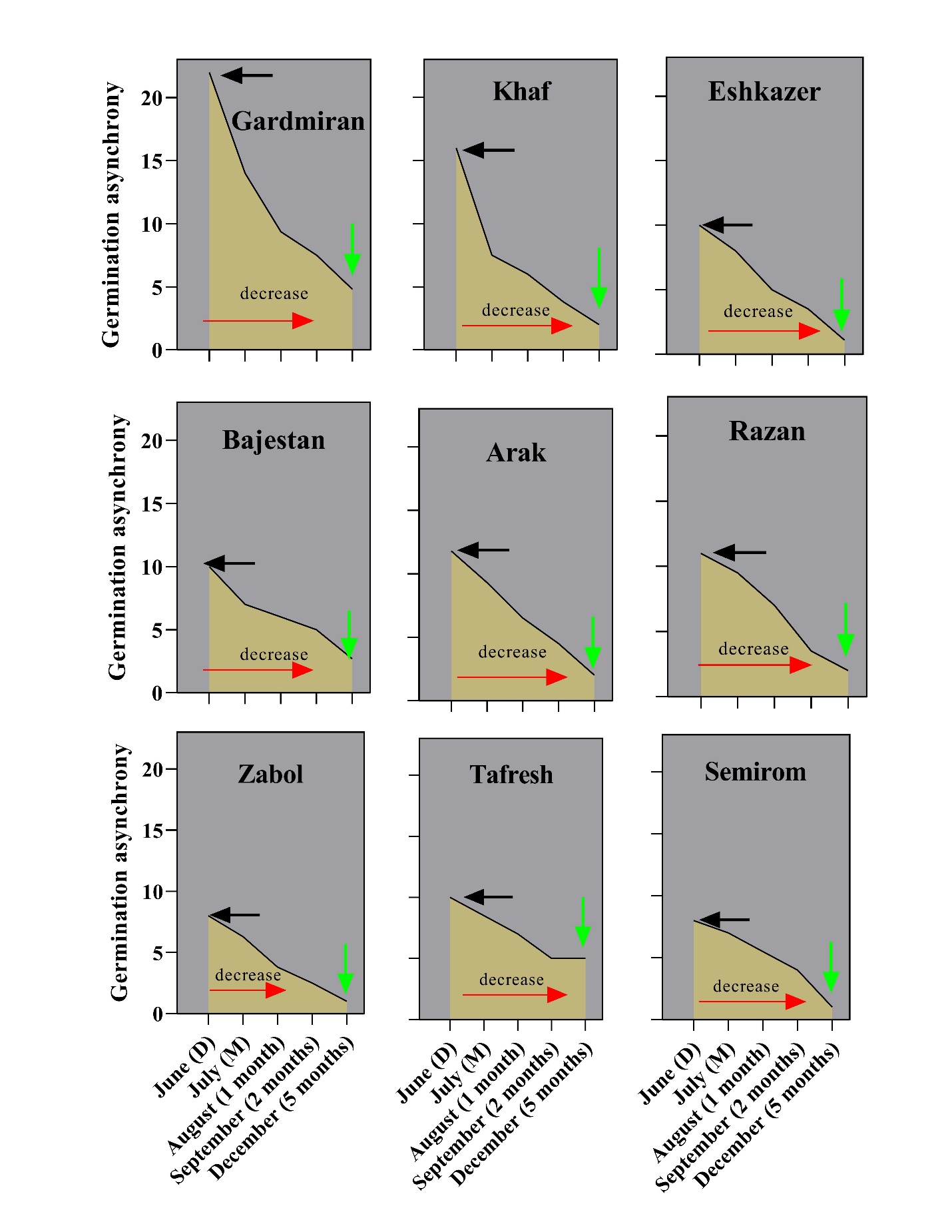


**Figure S7.** Graphical illustration showing synchrony of germination of different populations of *Nigella sativa*. Horizontal axis represents the intervals at which germination trials were performed (see Table 1). Vertical axis indicates synchrony of germination estimated by equation 5 (standard deviation of higher limit temperature that describes dormancy status). Note that the decreasing trend, the red arrows, show that synchrony or uniformity of germination is increasing. The black and green arrows show asynchronous and synchronous germination, respectively.


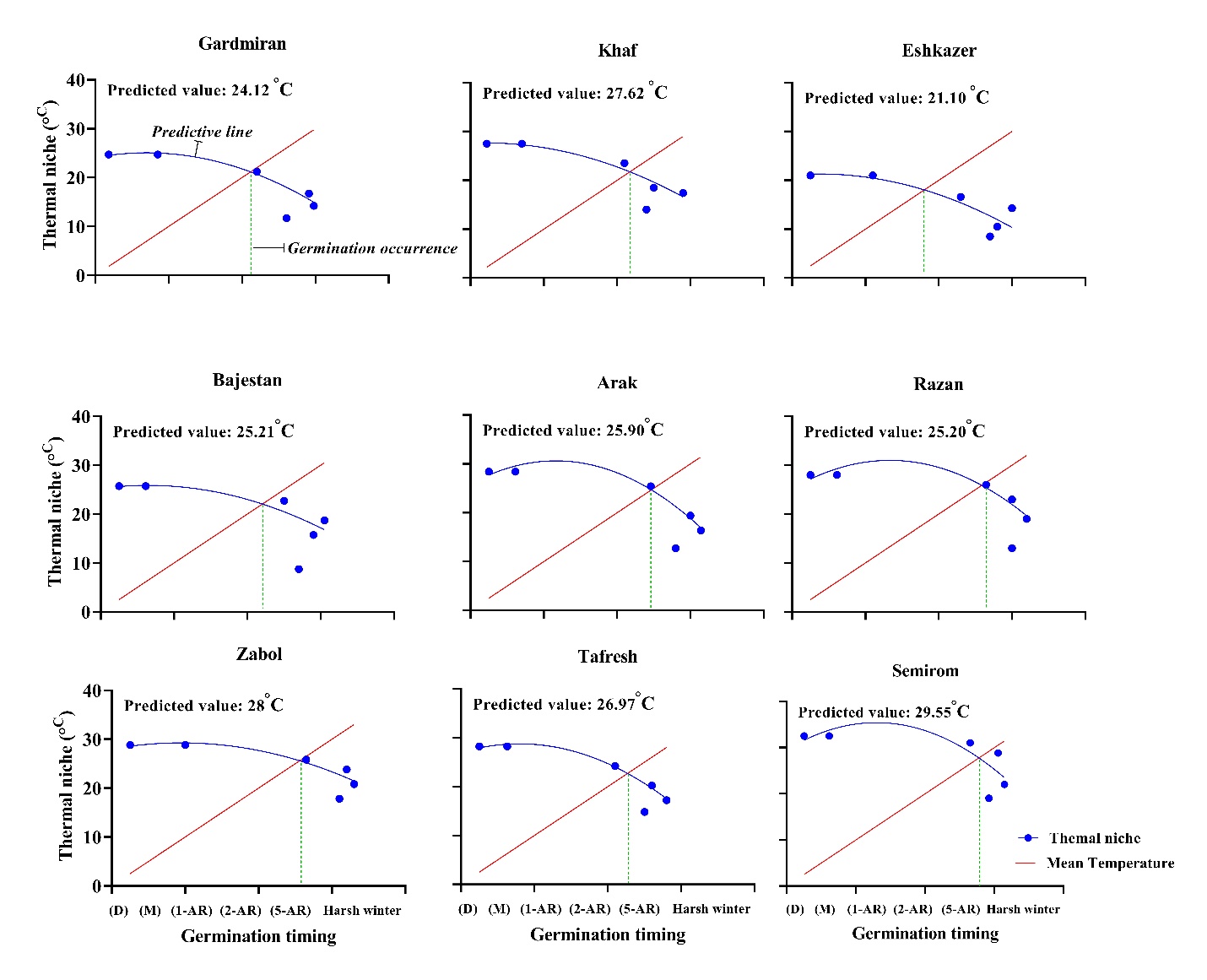


**Figure S8.** Graphical illustration showing germination timing in the field. The predicted values were estimated via non-linear regression. Blue lines show thermal niche and red lines indicate the mean temperature for each site and population. Germination occurs when blue line intersects red line. D, immature seeds; M, mature seeds; 1-AR, one month of afterripening; 2-AR, two months of afterripening; 5-AR, five months of afterripening.
